# Camera-based respiratory rate monitoring in unmodified neonatal intensive care – a cross-sectional study in a UK hospital

**DOI:** 10.1016/j.eclinm.2026.104175

**Published:** 2026-08-31

**Authors:** Alex Grafton, Lynn Thomson, Kathryn Beardsall, Joan Lasenby

**Affiliations:** a Department of Engineering, University of Cambridge, Cambridge, UK; b Department of Paediatrics, University of Cambridge, Cambridge, UK

**Keywords:** Neonatal, Respiratory rate, Non-contact monitoring, RGB-D, Computer vision, Biomedical signal processing

## Abstract

**Background:**

Camera-based methods have shown potential for respiratory rate measurement in neonatal intensive care. However existing work is tested on short recordings, which do not represent the range of environments present in the neonatal intensive care unit (NICU) or assess challenging conditions such as movement or poor visibility. This study evaluates a fully automatic respiratory rate measurement system over continuous 24-h periods in the unmodified NICU environment.

**Methods:**

In this single-centre cross-sectional study, 20 babies in the NICU in Addenbrooke's Hospital, Cambridge, UK were recorded using a colour + depth camera, each for around 24 h. Recordings took place between 11 July 2022 and 11 November 2025. Inclusion criteria were: pre-term and nursed in closed incubators; no specific exclusion criteria applied. Existing methods were used to automatically locate the baby, and we used a novel clustering method to extract a respiratory signal and detect the patient's breaths. The primary outcome was the measurement of respiratory rate using the camera, assessed by comparison with data from the clinical standard patient monitor, recorded concurrently. Further post-hoc analysis was conducted on clinical and demographic subsets. This trial is registered on clinicaltrials.gov, ID NCT04831242.

**Findings:**

After excluding intervals where the camera or baby was removed from the incubator, 380 h of valid recording from 20 participants remained. During non-intervention periods (338 h, 77.6% of all, 88.7% of valid), we configured the patient monitor signal quality indices to 57.9% valid measurement, a likely overestimate; the camera achieved 57.8% valid measurement with 1.44 breaths/minute agreement (mean error 0.42) averaged over all periods where both camera and monitor had valid data (136 h, 40.4% of non-intervention, 31.4% of all). Covering affects the valid time (63.6% when uncovered, 51.3% when covered), as do intervals where the baby is more active (61.4% when inactive, 53.5% when active). The error and valid time were worse when the monitor respiratory rate was below 35 breaths/minute (31.0% vs 57.8% valid, 1.98 vs 1.44 error) and more so below 25 breaths/minute (25.6% valid, 7.57 error), though data in this range was limited (0.2% of non-intervention) and the patient monitor may be unreliable.

**Interpretation:**

Respiratory rate measurement using cameras has potential for continuous use in the NICU, beyond previously studied short recordings, achieving mean absolute error (MAE) averaged across all data below 2 breaths/minute while providing valid data 57.8% of the time. The relationship between covering/activity and valid measurement time shows the importance of long recordings in the real-world NICU. The MAE and valid time was worse for babies under 1000 g, requiring continuous positive airway pressure, or when the respiratory rate is below 35 breaths/minute. Further work should focus on bradypnoea/apnoea detection, the aforementioned demographic groups, and results should be confirmed in a cohort with greater ethnic diversity.

**Funding:**

Rosetrees Trust, Stoneygate Trust, Isaac Newton Trust, EPSRC Impact Acceleration Account.

**Research in context**

**Evidence before this study**

We searched for relevant publications in PubMed published between 1 Jan 2010 and 1 December 2025, using the search terms: (neonatal OR newborn OR p(a)ediatric OR baby OR children) AND (“respiratory rate” AND (measurement OR extraction OR calculation) AND (remote OR camera OR imaging OR infrared OR depth OR non-contact), yielding 188 results. Work that did not directly measure respiratory rate, such as apnoea or distress detection, was excluded. Work that did not primarily focus on the neonates in intensive care, used simulators, or did not investigate contactless monitoring was also excluded. Numerous studies have measured respiratory rate in neonatal intensive care using RGB cameras, depth cameras, or radar. A range of methods for locating and extracting a respiratory signal are used, such as manual region selection, motion detection, or AI models for image processing. However, the vast majority are proof-of-principle studies using very short recordings, from 10 s to a few minutes, which cannot represent the range of conditions present in the real-world neonatal intensive care unit (NICU). Few studies included recordings longer than an hour, and those that do select short periods within this time to use for evaluation, excluding intervals where the patient is difficult to see or is moving. Just one study has used continuous recordings over several hours, though large portions are removed before analysis. No study has continued testing the method overnight. To demonstrate the potential application of the non-contact monitoring system, its performance must be presented in the context of the real-world NICU where movement and poor visibility are unavoidable.

**Added value of this study**

In contrast to previous studies, our camera-based respiratory rate measurement system is developed and tested using continuous 24-h recordings from 20 pre-term neonates in intensive care. The only excluded intervals are those where the baby or camera have been removed from the incubator, such as for kangaroo care, and no changes were made to the baby's care during the study. This allows us to test the system as though it were in continuous clinical use, rather than specific test cases. Using colour, depth and infra-red images, our method requires no manual interaction and functions in low light. Our method automatically identifies poor quality regions in the camera signal and patient monitor waveform, and we find that the agreement between camera and patient monitor is within two breaths per minute mean absolute error when approximately 60% of data from each is considered valid. In addition, the camera and patient monitor provide valid data at different times, so using both methods together increases the time for which a measurement is available (57.9%–75.3%). These results demonstrate the feasibility of using non-contact respiratory rate measurement continuously in the NICU, despite the challenges of poor visibility and movement. We also identified two subgroups where the method's performance is worse–patients under 1000 g, and those receiving continuous positive airway pressure (CPAP) respiratory support. A novel method is presented for identifying the respiratory signal combining computer vision and motion analysis.

**Implications of all the available evidence**

Our results demonstrate the feasibility of measuring a neonate's respiratory rate in the real-world NICU over 24-h periods, particularly regarding the use of depth cameras to detect movement through a blanket and to ensure continued measurement overnight. We also find that patients under 1000 g and those supported by CPAP should be strongly represented in further studies, as the method has worse performance on these patients.

## Introduction

Vital sign measurement is a key monitoring parameter in the neonatal intensive care unit (NICU). Respiratory rate monitoring is typically performed using thoracic impedance pneumography, where a high frequency current travels between ECG electrodes on the patient's chest. Expansion and contraction cause variation in the measured impedance, resulting in an oscillating signal representative of the patient's respiration.

However, ECG electrodes may damage new-born's fragile skin, and the array of equipment in the incubator impedes interaction with the baby and kangaroo care.^1^ Non-contact monitoring using cameras has been developed for vital sign measurement, including respiratory rate monitoring, in small studies by detecting movement of the baby's chest.

Beyond the non-contact benefits of camera monitoring, the opportunity for retrospective video analysis by clinicians provides additional advantages. A video of the baby may provide insights into the baby's respiratory pattern, asynchrony and paradoxical breathing, and respiratory effort. Furthermore, impedance pneumography is well-known to be susceptible to motion-based artefacts. While camera-based monitoring is similarly susceptible, the video information provides accompanying context to detect motion and confidently reject spurious measurements.

Non-contact respiratory rate measurement has been explored in multiple studies, primarily using RGB (red-green-blue colour), depth or thermal imaging, or radar. Initial work using RGB cameras required manually selected regions,^2^, ^3^, ^4^ though further studies have used motion detection^5^^,^^6^ and later deep learning models to process the visual scene.^7^, ^8^, ^9^ More recent work using RGB images includes mobile phones for instant on-demand measurements.^10^^,^^11^

Depth images are less frequently used; existing studies have required manually selected regions,^12^ which is infeasible for continuous use. Other work has used a combination of depth imaging and radar, though on a simulated baby.^13^ Alternative imaging methods include thermal imaging focussed on the subject's face^14^^,^^15^ or using motion detection,^16^, ^17^, ^18^ or radar.^19^, ^20^, ^21^

The majority of the existing work uses small datasets consisting of recordings as short as 10 s.^22^ Others^8^^,^^18^^,^^23^ use longer datasets but select time periods where the data is most amenable to respiratory rate measurement. The work of Villarroel et al.^9^ is the exception, where 400 h of video is used, though this is reduced to 140 h for analysis. The RGB-based work used by Huang et al.^24^ uses 16 h of video from neonatal care, but individual clips are around 20 min in length and the scene is illuminated throughout. These shorter recordings prove the principle of non-contact respiratory rate measurement, but do not offer insights into its continuous use in the real world.

The objective of this study is to assess the possibility of continuous, depth camera-based neonatal respiratory rate measurement in the unmodified NICU and compare it to the clinical standard patient monitor. We hypothesise that the depth camera is an effective measurement system due to its zero-light functionality, as incubators are frequently covered. We present a fully automated pipeline for non-contact respiratory rate measurement and evaluate it using data recorded in the unmodified NICU, including periods where the baby is in active or sleep states, or covered by blankets. Our recordings are up to 24 h long, to ensure that staff and parents cannot schedule their interactions with the baby around the recordings and must instead treat the system as a permanent part of care, including removing and replacing it without the supervision of the study team. Colour, depth and infra-red imaging are used to locate the baby even in low-light conditions, and the depth data is used to extract a respiratory signal via a novel signal processing method, and the signal is compared to the clinical patient monitor. We show that the camera can measure within two breaths/minute of the patient monitor with 60% valid measurement time, and can enhance existing measurement when used in conjunction, subject to further investigation in particular patient demographics.

## Methods

### Clinical study design

We conducted a single centre cross-sectional study, recruiting participants from the NICU at Addenbrooke's Hospital, Cambridge, UK. All NICU patients were eligible for the overall data recording study; for this work, a subset was selected based on the following inclusion criteria: born pre-term, nursed in a closed incubator, availability of the respiratory signal from the patient monitor, and with either no respiratory support, continuous positive airway pressure (CPAP), or high-flow nasal cannula (HFNC), or ventilation. The total number of participants in the overall study was 50, and 75 were approached (67% included). As we are assessing the feasibility of continuous respiratory rate measurement we are unable to calculate a target sample size a priori. Instead, the number of 20 participants was chosen to provide more data than any similar study in the literature; after data was collected and divided into subsets, further sample size analysis was undertaken.

Participants were recorded for 24 h with no changes to clinical care. Parents were able to remove their baby from the incubator for kangaroo care, and the camera was temporarily removed by clinical staff for complex procedures. An Azure Kinect (Microsoft, USA) RGB-D (RGB plus depth) camera was used, providing time-synchronised RGB, depth and infra-red (IR) images at 30 frames per second. The RGB resolution was 1280 × 720 pixels. The depth/IR images were captured at 640 × 576 pixel resolution and upsampled to match the RGB resolution during spatial alignment. The camera was placed outside the incubator, against the lid to prevent reflections and minimise the variation of the patient's distance from the camera. Patient monitor data was collected simultaneously, including the ECG, respiratory waveform, and measured respiratory rate. All data was recorded using custom software.

### Data pre-processing

Three recordings, not included in the 20 presented, were excluded due to particularly poor patient monitor data, which prevented manual time-synchronisation of the data sources (further synchronisation details and examples of poor quality data are given in the Appendix, Section 1). This data exclusion is due to failure of the clinical standard, not the camera, and may lead to over-estimation of the patient monitor's valid recording time. No recordings were excluded due to poor quality camera data.

The recordings were manually labelled based on the visual content. Data is Invalid if the baby is not present or the camera has been moved. Interventions, when the baby is observed and the measurement is less clinically significant, are separated. The remainder is labelled as covered/uncovered and active/inactive. Periods are labelled as covered if the baby's thorax is covered by bedding. Active periods include frequent movement of the baby's chest, or arm movement around the chest. These periods are labelled broadly, and there are inactive moments within active periods, and vice-versa. Each recording is divided into recording intervals, in which the intervention, covering and activity state do not change.

### Signal extraction

We locate the torso and extract a grid of depth signals. The signals are clustered and averaged based on their similarity and location, and the best is chosen to detect peaks and troughs and measure the signal quality. Precise details for implementation are contained in the Appendix.

#### Grid position

We use overlapping 20 s windows with 1 s steps. Pose estimation is used once per window to locate the baby's shoulders and hips, indicating the torso. The pose estimation model is based on HRNet-W32^25^^,^^26^ with the IIF-2 retraining and image fusion approach from our previous work.^27^

We create a 12 × 12 grid using the torso positions. The grid centre c whose x co-ordinate (left-right) is the mean joint x co-ordinate, and whose y co-ordinate is one third from the minimum to maximum y co-ordinate (closer to the shoulder than the hip). A square is centred on c with side length equal to the average torso diagonal ($d_{1}$ and $d_{2}$) multiplied by 0.8, divided into a 12 × 12 grid, and each grid square's mean depth gives 144 depth signals for each window.

#### Clustering

We use spectral clustering to identify and average similar signals. As the respiratory movement of the baby can be small, we wish to exclude unhelpful grid squares from the average, which could be noisy, or out-of-phase–potentially paradoxical breathing, which is common in neonates–and would reduce the signal amplitude. The precise details of the spectral clustering are given in the Appendix (Section 2). We perform this clustering with N_C_ = 6 clusters and the cluster's signal is the mean of those assigned to it. We also consider an alternate method using principal component analysis (PCA) and choosing the six most significant components.

#### Cluster scoring

Inspired by active pixel detection schemes^5^^,^^17^^,^^28^ we score each cluster's signal according to the fraction of its energy within 20–100 breaths/minute and choose the highest. The spectrogram is calculated using Welch's method^29^ with segment length 200 samples. Each segment's mean and standard deviation are normalised.

**Fig. 1:**
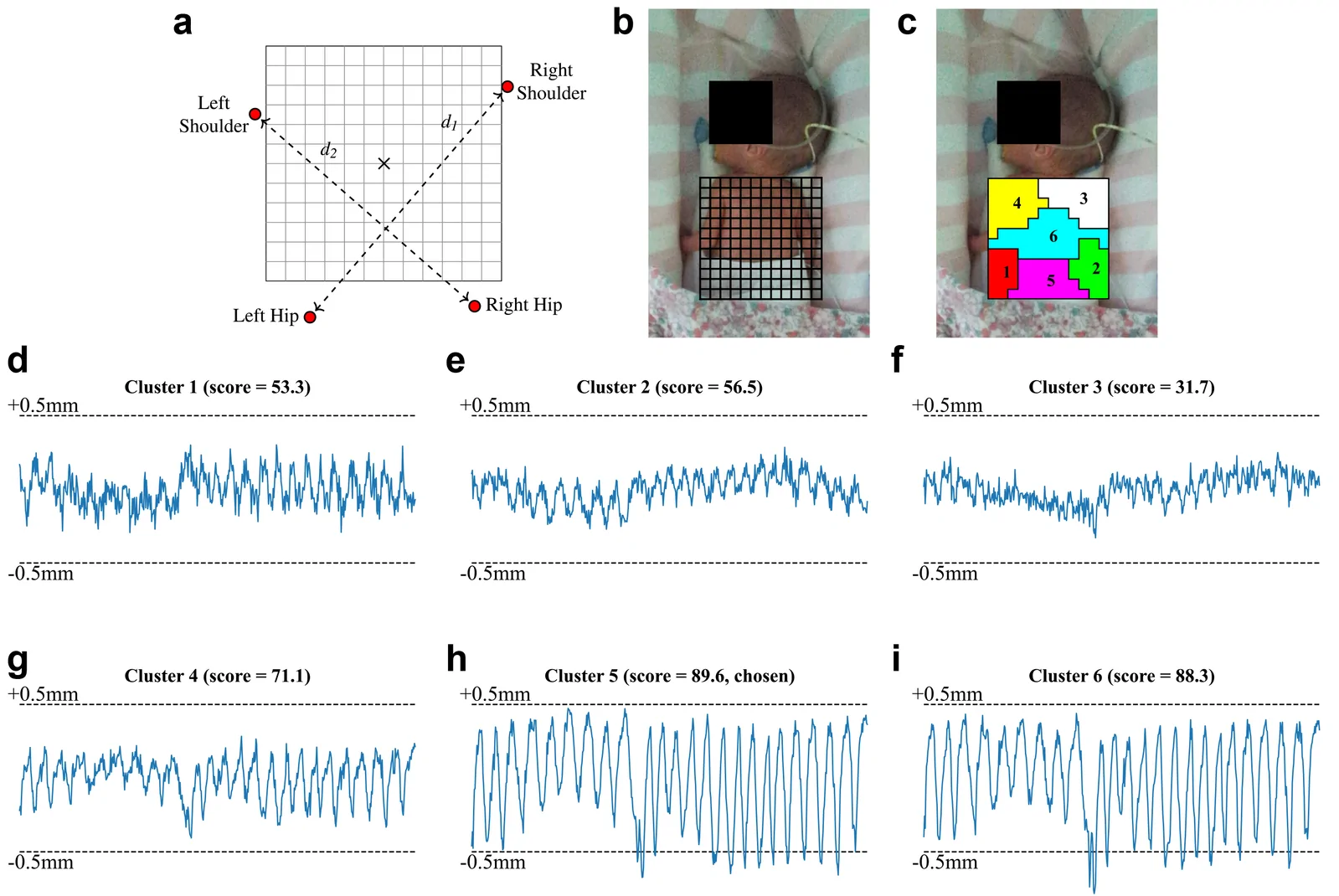
**Signal extraction from depth images. a**: Illustration of the grid position. *d*_1_ and *d*_2_ are the diagonal lengths; the square has side length 0.4 (*d*_1_ + *d*_2_), centred at the mean *y* co-ordinate and at one-third distance along the full range of *x* co-ordinates, from shoulder to hip. The square is divided into a 12 × 12 grid. **b**, **c**: The grid and clustered regions overlaid onto the image. **d–i**: signals obtained from each cluster. The dashed lines show ±0.5 mm. Cluster 5 is chosen due to its spectrogram energy distribution.

Fig. 1 illustrates the signal extraction process, showing the grid location, clustered regions and signals. As the region used is smaller than the baby's chest, it is able to adapt to the size of the patient without requiring modification based on the patient's age or weight.

### Breath selection

We find the signal's gradient using a local linear least-squares fit implemented with a 13-tap FIR filter. Gradients below 0.005 in magnitude are set to zero. Blocks of continuous positive or negative gradient whose length exceeds 5 samples are considered rises (positive) or falls (negative). Consecutive rises/falls with zeros between are merged, giving a sequence of alternating rises and falls.

We locate the peaks and troughs between rises and falls for each signal and apply a four sample minimum peak-trough distance and ten sample minimum peak–peak/trough–trough distance. We use the troughs as breath timings as periods with unusual respiratory patterns are more obviously characterised by the trough position.

Finally, breaths from each window are merged. We use every tenth window and combine troughs within 0.7 s. We correct out of phase signals by checking the signal value at the trough positions from the previous window; if these values are greater than the signal's mean (i.e. peaks), the signal is inverted.

### Evaluation

We use the patient monitor's waveform as ground truth to evaluate the camera's ability to extract a respiratory signal. We compare to the waveform instead of the patient monitor's respiratory rate as the precise calculation method is unknown, and differences in the method can cause considerable measurement error, described in the Appendix (Section 3).

The patient monitor marks inhalations and exhalations with notches on the waveform, coinciding with a lung icon appearing on the monitor. Peaks between notches denote breaths; we detect the notches and locate the maxima between each pair. Complete patient monitor signal processing details are presented in the Appendix (Section 4).

Camera and monitor breaths are matched using a maximised linear sum assignment, to find missing and extra breaths in the camera data. Precise details are provided in the Appendix (Section 5). To convert missed/extra breaths to measurement error, we use a 12-s window. Denoting a rectangular pulse from 0 to T as ${\text{rect}}_{T}\left(t\right)$, the times of extra breaths $e_{i}$ and missing breaths ${\;m}_{i}$, the error is:

$$error\left(t\right)=\sum_{i}\frac{60}{T}{rect}_{T}\left(t\;-\;e_{i}\right)\;-\;\sum_{i}\frac{60}{T}{rect}_{T}\left(t-m_{i}\right)$$

where T=12. The error signal is used to calculate the bias and mean absolute error. We also use a more traditional method, counting the number of breaths, including fractional breaths, within a sliding 12-s window. Calculations are repeated using window sizes from 6 to 18 s; we believe a 12 s window to be a suitable value by observing the patient monitor's rate measurement, which increases gradually, as it responds to a sharp jump in respiratory rate.

### Signal quality indices

As in the literature, we measure signal quality to indicate the measurement's reliability. Inspired by the work of Villarroel et al.,^9^ we consider two signal quality indices (SQIs), applied to both camera and monitor signals. Each SQI equals 0 if the signal is good and 1 otherwise. The SQIs are combined between overlapping windows and SQI types by setting the SQI to 1 if any overlapping SQI is 1. When evaluating the measurement accuracy, each breath's validity depends on the SQI during its time period. Missing/extra breaths are counted only if the breath's signal is valid, and the error is calculated over the valid time only. The Peak-Trough Amplitude SQI ensures that consecutive peaks and troughs have similar vertical distance, and the Shape SQI ensures that the breath follows an up-down or down-up trajectory.

Selecting the thresholds for these SQIs is somewhat arbitrary, particularly for the patient monitor signal as the true respiratory rate is unknown. Existing literature has estimated the time for high patient monitor signal quality at around 40%,^9^^,^^30^ though we expect the time for which breaths are correctly found (even with a poor quality signal) to be higher.

We select SQI thresholds to give around 60% validity during non-intervention periods, which provide the main results in this paper. We also vary each SQI threshold to show the relationship between measurement error and valid time. Precise implementation details and examples are provided in the Appendix (Section 6).

### Comparison to existing literature

To compare our method to existing work, we recreate three other methods using our dataset. These methods are chosen because they use depth imaging or use particularly large datasets. As we focus on 24-h recordings, we note that the patient is barely, if at all visible during the night making methods that require RGB-specific features unsuitable. These methods were originally tested on data where babies are unclothed and mostly still and require specific annotations. As such, we choose a continuous uncovered, inactive, 1-h period from each participant to manually annotate. Although well-illuminated intervals are chosen to make manual annotation possible, we test methods that could feasibly work in darkness. For six participants, no such 1-h period exists. Manual annotations are created at each second. The compared methods are:

• Villarroel et al. (2019)^9^: The signal is extracted from an ellipse around the skin area and processed using the moving average curve^31^^,^^32^ or boxed slope sum function^33^ (BSSF) methods, denoted Ellipse, MAC and BSSF.
• Cenci et al. (2015)^12^: Measurement points are selected on the chest, and filtering/peak finding identifies breaths, denoted Measurement Points and Peak Finding.
• Cobos-Torres et al. (2018)^34^: The signal is extracted from a small rectangle, and the peak of the discrete Fourier transform (DFT) within a range 30–60 breaths/minute provides the respiratory rate, denoted Rectangle and DFT Peak (30).

Each method is tested using RGB, depth and IR images. The DFT Peak method is also tested with a wider band-pass filter (20–100 breaths/minute), denoted DFT Peak (20). We also test a combination of the above signal extraction methods with our signal processing method. Complete details of the adjustments made to each method are provided in the Appendix (Section 7).

### Statistical analysis

Results are reported as the mean absolute error (MAE), bias (the mean error, where positive bias indicates that the camera estimates a higher respiratory rate) and percent valid measurement time. To estimate confidence intervals, we estimate the distribution for each participant then bootstrap from those distributions. Per-participant distributions are estimated using moving-block bootstrapping,^35^ which represents a recording as *N* blocks of length *B*, with blocks sampled from the actual recording. We choose this method as it accounts for correlation in the time-series data. The block length (in samples) is $\sqrt{n}$ or n/2, whichever is lower, where n is the number of samples for that participant/subset; these lengths are of the same order of magnitude as the optimal block length given by the spectral density method.^36^^,^^37^ The n/2 length is rarely selected and arises from very small subsets. Subset analysis and testing is conducted by the same method, where the number of participants in each bootstrapped sample is the number of participants in that subset.

The expected time spent in each particular covering/activity state was not known a priori so sample-size calculations were conducted post-hoc. The effect of sample size on confidence intervals was computed by varying the number of samples in the bootstrap. Post-hoc subset analysis was conducted by dividing the participants by gestational age, weight, sex, ethnicity and level of respiratory support. The number of samples in the bootstrap was the number of participants in each subset. Recordings were divided by the patient's covering and activity, day/night and current respiratory rate and the analysis repeated; in this case, n, used to calculate the block length, was the number of samples in that participant's recording matching the subset. Subsets with significantly different results are identified using one-sided tests on the null distributions. Main results are also repeated with the 12-s window size being varied between 6 and 18 s.

Analysis was conducted using Python (version 3.11.1), NumPy (2.4.4), SciPy (1.15.2) and R (4.6.0).

### Ethics

The study received approval from the UK Health Research Authority (North West—Preston Research Ethics Committee, reference number 21/NW/0194, IRAS ID 285615) and followed the ethical guidelines of the 1964 Helsinki Declaration. Written, informed consent was received from each participant's parent/guardian prior to any study procedures.

### Role of the funding source

The funder of the study had no role in study design, data collection, data analysis, data interpretation, or writing of the report.

## Results

Table 1 summarises the study participants and data collection, divided by validity, intervention, activity and covering. A total of 434.6 h of video was recorded from 20 participants, of which 380.6 h (87.5%) were considered valid. Data from all 20 participants were included in the valid subset. Histograms of respiratory rates during each period and the length of recording from each participant are provided in the Appendix (Sections 8 and 9). Table 2 lists the valid time percentages and error measurements for both evaluation methods. We list the raw valid percentage, and the percentage if continuous 12-s intervals are required, but 18-s gaps are permitted (one measurement every 30 s). During non-intervention periods, the camera and monitor have similar valid times, but the camera is much worse during interventions. When the baby is uncovered or inactive, the camera has greater valid time compared to the patient monitor (63.6% vs 57.7% and 61.4% vs 60.1%) but is lower when the baby is covered (51.3% vs 58.1%) or active (53.5% vs 55.3%). The time when at least one measurement is valid is 75.3% for non-intervention data. The patient monitor's valid time is unaffected by covering.

**Table 1:** Summary of participants and recorded data.

Cohort	
GA at birth (weeks + days)	27 + 3 ± 17 d (23 + 1, 32 + 1)
GA at recording (weeks + days)	30 + 4 ± 15 d (27 + 1, 34 + 6)
Age at recording (days)	21 ± 14 (3, 47)
Birth weight (g)	950 ± 305 (500, 1555)
Recording weight (g)	1162 ± 265 (625, 1630)
Sex	11 Male (55%)/9 Female (45%)
Ethnicity	16 White (80%)/4 MEB (20%)
Respiratory Support	1 SV, 11 HFNC, 7 CPAP, 1 Vent
Recording time	
Total (hours)	434.6 (100.0%)
Invalid Data (hours)	54.0 (12.4%)
Intervention (hours)	43.2 (9.9%)
Non-Intervention (hours)	337.5 (77.6%)
Non-intervention (NI) recordings	
Uncovered, Inactive (hours)	92.5 (27.4% of NI, 21.3% of Total)
Uncovered, Active (hours)	84.7 (25.1% of NI, 19.5% of Total)
Covered, Inactive (hours)	89.4 (26.5% of NI, 20.6% of Total)
Covered, Active (hours)	70.8 (21.0% of NI, 16.3% of Total)

Participants' gestational age (GA) is given as weeks plus days. Ethnicity is divided into white and minority ethnic background (excluding white minorities) (MEB) due to the higher proportion of white patients in the neonatal unit. SV = self-ventilating, HFNC = high-flow nasal cannula, CPAP = continuous positive airway pressure, Vent = ventilated. Where appropriate, values are given as mean ± 1 standard deviation, (maximum, minimum). Invalid data is when the baby is not present in the image, or the camera is pointed away from the incubator. Interventions are times when someone was physically interacting with the baby.

**Table 2:** Percentages of time with valid signal quality, as determined by our signal quality indices, for the camera and patient monitor in 24-h recordings, and measurement error when both are valid.

All participants									
Subset	Validity					Breath matching		Rate comparison	
	Monitor	Camera	Either	Both	Agree	MAE	Bias	MAE	Bias
All	57.0 (44.1) (51.1–62.3)	56.1 (46.6) (53.4–63.9)	74.0 (63.8) (72.8–78.9)	39.0 (26.9) (34.1–46.1)	65.0 (61.2–67.8)	1.52 (1.21–1.90)	0.46 (0.11–0.82)	1.58 (1.21–1.97)	0.38 (0.05–0.68)
Intervention	50.1^∗^ (31.8) (46.3–53.8)	42.5^∗∗∗^ (27.6) (38.4–48.1)	64.6^∗∗∗^ (44.1) (62.5–68.3)	28.0^∗∗∗^ (15.3) (24.4–31.6)	63.4 (61.2–64.7)	2.42^∗∗∗^ (1.77–3.22)	0.83^∗^ (0.23–1.43)	2.66^∗∗^^∗^ (1.61–3.62)	0.85^∗∗^ (0.28–1.28)
Not Intervention	57.9 (45.7) (51.5–63.6)	57.8 (49.0) (55.1–66.3)	75.3 (66.4) (73.9–80.4)	40.4 (28.4) (35.1–48.2)	65.2 (61.1–68.4)	1.44 (1.16–1.80)	0.42 (0.07–0.79)	1.51 (1.18–1.87)	0.35 (0.02–0.65)
Uncovered	57.7 (44.9) (50.2–65.1)	63.6 (55.9) (60.7–69.8)	76.5 (68.3) (74.0–81.7)	44.8 (32.5) (37.8–52.7)	68.3 (63.6–71.7)	1.48 (1.08–1.99)	0.58 (0.14–1.05)	1.43 (1.06–1.92)	0.59 (0.30–0.96)
Covered	58.1 (46.6) (52.1–63.7)	51.3^∗^ (41.4) (49.2–62.6)	73.9 (64.2) (73.3–79.8)	35.6 (23.8) (31.4–44.9)	61.7^∗^ (57.9–65.7)	1.39 (1.17–1.65)	0.21 (−0.08–0.49)	1.62 (1.28–1.95)	−0.02† (−0.43–0.34)
Inactive	60.1 (50.0) (52.2–66.6)	61.4 (54.7) (56.8–71.8)	78.0 (71.7) (76.5–83.4)	43.5 (33.1) (36.6–53.3)	65.5 (59.9–70.2)	1.20 (0.99–1.49)	0.25 (−0.10–0.56)	1.28 (1.01–1.63)	0.19 (−0.18–0.46)
Active	55.3 (40.7) (49.6–61.0)	53.5^∗^ (42.4) (51.3–61.0)	72.0^∗^ (60.1) (70.4–77.5)	36.9 (22.9) (32.4–43.1)	64.9 (61.6–66.8)	1.77^∗^ (1.37–2.20)	0.66 (0.23–1.11)	1.90^∗^ (1.41–2.32)	0.62^∗^ (0.20–1.02)
Uncovered, inactive	60.7 (50.9) (50.8–70.0)	69.5^†††^ (64.2) (66.2–75.3)	80.2^†^ (75.2) (76.8–85.5)	50.1^†^ (39.9) (40.9–59.7)	69.9^†^ (63.9–74.5)	1.21 (0.91–1.64)	0.30 (−0.14–0.67)	1.11^††^ (0.92–1.42)	0.37 (0.20–0.59)
Uncovered, active	54.4 (38.4) (48.1–60.8)	57.2 (46.9) (54.3–64.0)	72.5^∗^ (60.8) (70.2–78.2)	39.0 (24.5) (33.3–45.3)	66.5 (62.9–68.8)	1.85^∗^ (1.28–2.46)	0.94^∗∗^ (0.39–1.50)	2.01^∗^ (1.34–2.64)	0.98^∗∗∗^ (0.42–1.49)
Covered, inactive	59.4 (49.2) (51.9–65.0)	53.1 (44.9) (49.2–67.8)	75.9 (68.1) (75.9–81.8)	36.7 (26.0) (31.7–48.0)	60.8^∗^ (55.8–66.7)	1.21 (1.05–1.40)	0.17 (−0.15–0.50)	1.54 (1.14–1.99)	−0.11 (−0.65–0.37)
Covered, active	56.5 (43.4) (50.2–62.8)	49.1^∗∗^ (37.0) (47.7–57.7)	71.4^∗^ (59.3) (69.9–77.7)	34.3^∗^ (21.1) (30.3–41.6)	62.9 (59.5–65.2)	1.63 (1.31–1.98)	0.27 (−0.02–0.56)	1.75 (1.36–2.09)	0.13 (−0.15–0.40)

Selected Subsets (Significant MAE)				
Subset	Size	MAE	Camera valid %	Method
Ethnicity—MEB	N = 4	1.07^†††^ (0.81–1.77)	64.4	Breath matching
Respiratory support—CPAP	N = 7	1.89^∗^ (1.48–2.46)	51.8^∗^	Breath matching
Weight–<1000 g	N = 5	1.97^∗∗^ (1.28–2.92)	56.2	Breath matching
Night—covered	82.5 h	1.84^∗∗∗^ (1.34–2.31)	52.2	Rate comparison
Night—covered, inactive	53.3 h	1.77^∗∗∗^ (1.17–2.53)	52.8	Rate comparison
Respiratory rate <25	3.0 h	7.57^∗∗∗^ (5.53–12.53)	25.6 ^∗∗∗^	Rate comparison
Respiratory rate 25–35	24.2 h	1.98^∗^ (1.42–4.52)	31.0 ^∗∗∗^	Rate comparison

Validity entries are given as X (Y), where X only requires that the signal is valid, no matter how short the interval (used for breath matching), and Y requires continuous 12-s valid intervals but allows gaps of 18 s, ensuring one measurement every 30 s (used for rate comparison). Agree is the percentage of time where the camera and patient monitor are both valid or both invalid. Errors are given as MAE (mean absolute error) and bias (mean error), both in breaths/minute. Positive bias indicates that the camera measures a higher rate than the monitor. Errors are given using breath matching and measured respiratory rate comparison. 95% confidence intervals are given; for significant results, ∗, ∗∗, and ∗∗∗ indicate that the method performs worse on this subset (further from zero error or lower validity) at the p = 0.05, 0.005 and 0.0005 levels respectively, and †, ††, and ††† indicate that the method performs better on this subset at the same levels. Intervention and Non-Intervention are tested against All; others are tested against Non-Intervention.

The mean absolute errors are below 2 breaths/minute in non-intervention data, with upper confidence intervals below 2.5 breaths/minute. The errors are typically higher using the rate comparison method, possibly due to breath positions being at slightly different time indices. Covering reduces the valid time; activity reduces the valid time and increases the MAE (p < 0.05). When the baby is inactive, covering does not significantly alter the MAE but greatly reduces the valid time (50% vs 37% both valid, 70% vs 53% camera valid).

**Fig. 2:**
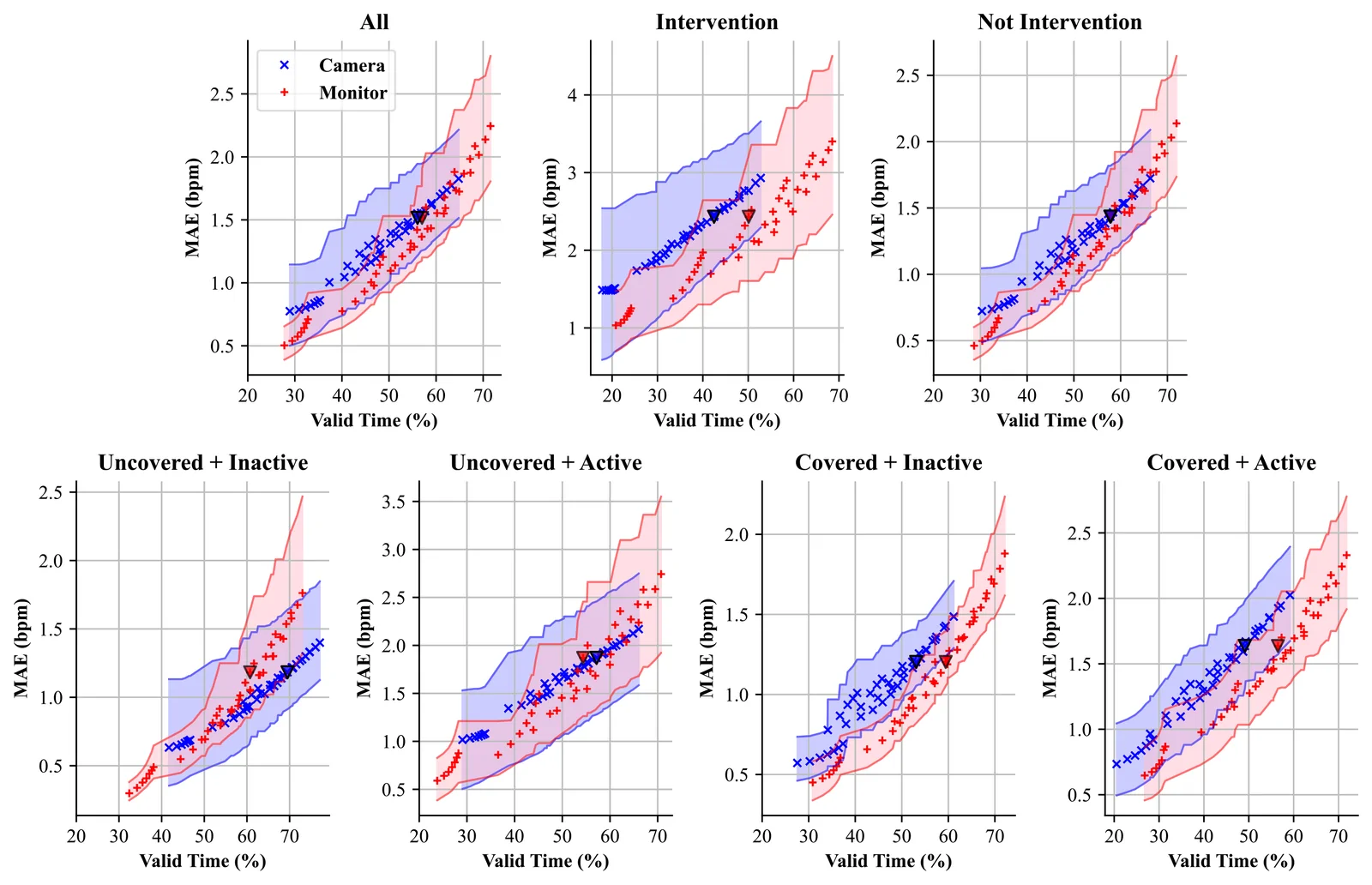
**Relationship between valid time and mean absolute error (MAE) as signal quality index (SQI) thresholds are varied, divided between covering and activity.** The monitor data points are generated by holding the camera thresholds constant and varying the monitor SQI thresholds, and vice-versa. The error is only calculated over the data which is valid for both sources. The triangle icons are for the thresholds used in the rest of this work. For 'Not Intervention' the triangles overlap. bpm = breaths per minute. 95% confidence intervals (CIs) shown as shaded regions. The CIs are slightly modified to ensure that the upper CI always increases from left-right, and the lower CI decreases from left-right; this marginally widens the interval to improve readability.

The relationship between MAE and valid time for the monitor and camera are shown in Fig. 2. In each case, the SQI thresholds for one method are held constant and the others are varied, altering the MAE and valid time. There is a positive relationship between MAE and valid time, indicating that the SQIs are removing invalid data. The clustering method outperforms the PCA signal extraction method in MAE, bias and valid time in almost all metrics–complete results are in the Appendix (Section 9) along with results by participant.

The results are also grouped by ethnicity, respiratory support, age, weight, sex, and day/night in the Appendix (Section 9, Tables S7 and S9). Significant results are described here. For non-intervention data (MAE = 1.44), the MAE is significantly improved for patients from ethnic minority backgrounds (1.07, p < 0.0005, N = 4), worse for patients supported by CPAP (1.89, p < 0.05, N = 7) and those weighing under 1000 g at the time of recording (1.97, p < 0.005, N = 5). During the night, the MAE is worse for covered patients (1.84 night, 1.39 day, p < 0.0005), particularly when also inactive (1.77 night, 1.25 day, p < 0.0005) when evaluated by comparing the respiratory rate. In all cases, the upper MAE confidence interval is below 3 breaths/minute.

The MAE is significantly worse when the estimated patient monitor respiratory rate is below 25 (MAE = 7.57, p < 0.0005, 95% CI 5.53–12.53). This subset corresponds to 0.2% of the overall data, explaining the particularly wide confidence intervals. The MAE is also worse in the 25–35 breaths/minute range (1.98 vs 1.51), which corresponds to just 3.8% of the overall dataset, and also has wide confidence intervals (1.42–4.52). The MAE for the remaining ranges is not significantly different from the overall result; complete values are given in the Appendix (Section 9, Table S8). The valid measurement times are significantly lower for both patient monitor and camera below 35 breaths/minute; 25.5% and 25.6% respectively at <25 bpm during non-intervention periods, and 34.3% and 31.0% at 25–35 bpm. All results, by activity and covering, are given in the Appendix (Section 9, Table S9).

All main results are repeated with varying window lengths used for evaluation (currently 12 s). Decreasing window size leads to increasing error–MAE = 1.69 at 6-s windows and MAE = 1.34 at 18-s windows for non-intervention data (breath matching). These values are within the confidence intervals found previously. Using estimated rate comparison, the non-intervention MAE rises significantly from 1.65 (12-s windows) to 2.31 (6-s windows) (p < 0.0005). The greatest change is the uncovered, active case which rises from 2.19 (12-s windows) to 3.01 (6-s windows). The complete results table is given in the Appendix (Section 9, Table S10).

Confidence intervals as a function of the number of study participants are given in the Appendix (Section 11). Excluding interventions, the uncovered, active subset has the widest confidence intervals; to reduce the upper bias 95% confidence intervals to within 0.5 breaths/minute of the estimated value, we estimate an increase to 27 participants would be required; given that the time in the subset is not fixed between patients, 40 participants would be more realistic.

Scores from other methods are presented in Table 3, using data from a 1-h period from each recording where the baby is uncovered and mostly still. Our method achieves the best MAE (0.54 bpm–all others are significantly worse, p < 0.0005), though the method is adapted to our dataset. Certain methods' bias measurements are not significantly worse, though a larger MAE indicates a wider range of errors. Other high-performing methods are ellipse signal extraction plus our signal processing (MAE = 1.92 bpm) and measurement points signal extraction plus our signal processing (MAE = 2.09 bpm), both using the depth data. RGB images produce poor respiratory rate estimates, as does pure peak finding. Our modification to widen the filter's passband in the DFT peak method improves results, likely due to the wider range of respiratory rates in our dataset.

**Table 3:** Comparison of our respiratory rate measurement method with others.

Extraction	Image	Processing	Breath matching		Rate calculation	
			MAE	Bias	MAE	Bias
Ours	Depth	Ours	0.54 (0.44–0.69)	0.14 (−0.08–0.34)	0.86 (0.55–1.31)	0.29 (0.03–0.72)
Measurement points	Colour	Peak Finding	18.81^∗∗∗^ (13.71–24.25)	18.10^∗∗∗^ (12.66–23.82)	24.17^∗∗∗^ (17.65–31.52)	23.13^∗∗∗^ (16.05–30.83)
Measurement points	Colour	Ours	10.07^∗∗∗^ (7.33–13.33)	1.99^∗∗∗^ (−3.13–7.91)	13.11^∗∗∗^ (9.74–17.31)	1.75^∗∗∗^ (−5.17–9.76)
Measurement points	Depth	Peak Finding	7.53^∗∗∗^ (3.89–12.03)	7.35^∗∗∗^ (3.66–11.90)	8.83^∗∗∗^ (4.28–14.18)	8.44^∗∗∗^ (3.73–13.92)
Measurement points	Depth	Ours	2.09^∗∗∗^ (1.49–2.80)	0.20 (−0.68–1.10)	2.40^∗∗∗^ (1.59–3.33)	0.13 (−1.09–1.21)
Measurement points	Infra-red	Peak Finding	8.65^∗∗∗^ (5.90–11.85)	8.30^∗∗∗^ (5.42–11.64)	10.72^∗∗∗^ (7.11–14.98)	10.19^∗∗∗^ (6.34–14.65)
Measurement points	Infra-red	Ours	3.81^∗∗∗^ (2.71–5.27)	2.27^∗∗∗^ (0.80–4.08)	5.08^∗∗∗^ (3.37–7.33)	2.96^∗∗∗^ (0.75–5.66)
Rectangle	Colour	DFT Peak (30)	–	–	20.54^∗∗∗^ (15.20–26.12)	−18.01^∗∗∗^ (−25.06 to −10.62)
Rectangle	Colour	DFT Peak (20)	–	–	22.12^∗∗∗^ (17.61–26.59)	−16.12^∗∗∗^ (−22.82 to −8.99)
Rectangle	Depth	DFT Peak (30)	–	–	15.58^∗∗∗^ (10.37–21.64)	−12.96^∗∗∗^ (−20.16 to −6.34)
Rectangle	Depth	DFT Peak (20)	–	–	6.91^∗∗∗^ (5.68–8.32)	−2.04^∗∗∗^ (−3.39 to −1.01)
Rectangle	Infra-red	DFT Peak (30)	–	–	15.66^∗∗∗^ (10.69–21.51)	−13.17^∗∗∗^ (−20.05 to −6.79)
Rectangle	Infra-red	DFT Peak (20)	–	–	7.38^∗∗∗^ (6.34–8.57)	−2.25^∗∗∗^ (−3.67 to −1.05)
Ours	Depth	DFT Peak (20)	–	–	5.39^∗∗∗^ (4.52–6.35)	−0.45 (−1.26–0.20)
Ellipse	Colour	MAC	13.31^∗∗∗^ (11.72–14.91)	−0.54^∗∗∗^ (−6.18–5.15)	17.54^∗∗∗^ (14.98–20.54)	−2.34^∗∗∗^ (−10.59–5.68)
Ellipse	Colour	BSSF	11.61^∗∗∗^ (9.63–14.18)	2.66^∗∗∗^ (−2.45–8.07)	15.50^∗∗∗^ (12.82–18.98)	2.69^∗∗∗^ (−4.74–10.00)
Ellipse	Colour	Ours	13.44^∗∗∗^ (10.28–16.70)	−10.15^∗∗∗^ (−14.91 to −5.08)	18.08^∗∗∗^ (13.47–22.84)	−14.10^∗∗∗^ (−20.84 to −6.89)
Ellipse	Depth	MAC	4.53^∗∗∗^ (3.22–6.06)	−0.56^∗∗∗^ (−2.41–1.07)	5.98^∗∗∗^ (4.13–8.22)	−1.18^∗∗∗^ (−3.66–1.04)
Ellipse	Depth	BSSF	3.95^∗∗∗^ (3.15–4.93)	−1.83^∗∗∗^ (−3.26 to −0.43)	5.21^∗∗∗^ (4.13–6.52)	−2.22^∗∗∗^ (−4.12 to −0.45)
Ellipse	Depth	Ours	1.92^∗∗∗^ (1.12–3.09)	−1.18^∗∗∗^ (−2.21 to −0.41)	2.45^∗∗∗^ (1.39–4.02)	−1.28^∗∗∗^ (−2.69 to −0.28)
Ellipse	Infra-red	MAC	9.30^∗∗∗^ (6.11–13.03)	4.64^∗∗∗^ (0.58–9.22)	12.44^∗∗∗^ (8.36–17.13)	5.65^∗∗∗^ (−0.30–11.89)
Ellipse	Infra-red	BSSF	6.68^∗∗∗^ (4.56–9.32)	−0.05 (−3.82–4.10)	9.02^∗∗∗^ (6.13–12.59)	−0.13 (−5.33–5.60)
Ellipse	Infra-red	Ours	3.15^∗∗∗^ (2.14–4.24)	−0.36^∗^ (−1.92–1.15)	4.12^∗∗∗^ (2.65–5.71)	−0.52 (−2.64–1.59)

Other signal extraction methods are Measurement Points,^12^ a small rectangle on the baby's chest,^34^ or an ellipse around the skin area.^9^ Each method is tested using each channel; for the colour image, we test each colour channel individually and choose the best. We also multiply each signal by −1 and re-evaluate, choosing the best. The processing methods are Peak Finding,^12^ the peak of the discrete Fourier transform (DFT) after applying either a 30–60 bpm filter, or a 20–100 bpm filter (DFT Peak),^34^ the Moving Average Curve (MAC),^31^^,^^32^ Boxed Slope Sum Function (BSSF).^33^ We also test some methods in combination with our own (Ours). Breath matching scores are not provided for the DFT Peak method as it directly calculates the respiratory rate instead of providing individual breaths. ∗, ∗∗, and ∗∗∗ indicate that a method performs worse than ours (MAE or bias further from zero) at p = 0.05, 0.005, and 0.0005 levels respectively. Ranges indicate 95% confidence intervals.

## Discussion

We have presented a complete pipeline for neonatal respiratory rate calculation, requiring no manual configuration, evaluated on 20 24-h recordings in the NICU. No data was excluded due to the baby's activity or covering, so reported valid measurement times represent continuous NICU use during the recording. During non-intervention periods, the overall mean absolute error between the camera and patient monitor is below 2 breaths per minute.

The uncovered, inactive patient represents the ideal imaging conditions. The active, covered period is the worst case, with the valid measurement time decreased from 70% to 49%, so including these periods is necessary for assessing clinical viability. We suggest that this is due to the blanket distributing the effect of movement across the patient's body and hiding respiratory motion. The patient monitor's valid time is less dependent on conditions, varying from 61% to 57%. Further analysis, presented in the Appendix (Section 10), shows that movement affects the camera's measurement more than the monitor; the monitor is susceptible to other artefacts, such as detecting the heart rate.

Periods of invalid signals are excluded using two SQIs, ensuring that we only measure the camera's accuracy against reliable ground truth. There is therefore a natural conflict of interest, in that we are effectively able to choose when to compare the two measurement methods; we have not come across existing work that directly addresses this. We have intentionally configured SQI thresholds such that the patient monitor's valid time is around 58%, which exceeds that found in the literature^9^^,^^30^ by around 1.5× . This may result in poor quality signals being included in the measurement, but as the monitor is treated as a perfect ground truth, this only affects the reported accuracy of the camera. As we also have control over the camera's valid measurement time, the statement that the camera and monitor have the same valid measurement time is not itself a conclusion, but rather that the camera and monitor agree within 2 breaths/minute (breath matching) with the upper 95% confidence intervals within 2.5 breaths/minute when they both measure valid data around 60% of the time. Stating higher or lower valid measurement times would change the agreement measurement, as shown in Fig. 2.

The 20 participant dataset has been divided into subsets based on the ethnicity, respiratory support, gestational age, weight and sex of the participant. The CPAP and under 1000 g subsets had worse MAE than overall; of the five participants weighing under 1000 g, 2 had CPAP and 3 had HFNC, suggesting that these are separate factors. For these smaller patients, the monitor and camera valid times were both lower than average, despite spending more time in the uncovered state (76% vs dataset average 53%). We instead suggest that the lower amplitude of breathing motion for smaller patients explains this result. For the CPAP patients, there is no noticeable difference in the overall time spent in each covering and activity state. Instead, we hypothesise that the shape of chest wall movement of patients with CPAP may be different, particularly given that we observe the exterior surface, rather than the lungs themselves. For these two significant subsets, overall non-intervention MAE values still fall below 2 breaths/minute with upper confidence intervals below 3 breaths/minute.

A supposed advantage of using a depth camera is its performance in low- and zero-light conditions. Using the breath matching evaluation method, there are no significant differences in measurements taken during day or night shifts except for an improvement in bias for covered, active patients at night. However, when comparing the estimated respiratory rates, the MAE is significantly higher for covered patients at night, though the upper confidence interval is still within 3 breaths/minute and the overall MAE is not significantly higher. The most significant increase is for covered, inactive patients; the pose estimation may be less accurate given that they are more difficult to detect from depth images when covered. Disagreement between the two evaluation methods occurs when the same number of breaths are detected, but at different times; the monitor and camera measure movement below and above the blanket respectively, which combined with less accurate detection, leads to disagreement.

We have considered day-to-day respiratory rate estimation in this work, suitable for detecting trends that may indicate normal development or potential infection. Detecting acute changes—the other key functionality of the patient monitor—such as brady/tachypnoea is not considered. Examining measurement error by rate, we find the MAE between the camera and monitor is below 2 breaths/minute at all respiratory rate ranges except <25 (though with a large confidence interval for 25–35 breaths/minute, partly due to the small amount of data for this range). Below 25 breaths/minute, the MAE is much worse - 7.57 - though this time accounts for just 0.2% of the data, as it is far below the typical neonatal physiological range. Here, our use of the patient monitor as gold standard data breaks down; we use the patient monitor data to divide into ranges, and the monitor is notoriously inaccurate at low rates in both neonates and adults^38^, ^39^, ^40^; checking the camera's false positives is particularly difficult due to the need to manually review camera data, which may be dark. Capsule pneumography may provide a superior ground truth^39^ with less patient impact than CO2 measurement, and further work should target recruitment of patients specifically at-risk of apnoea; it should be noted that the capsule will be visible to the camera and may affect measurement.

Our method outperforms other methods in the literature, though some alterations were needed to adapt them to our dataset. Methods using RGB images perform poorly compared to their original work. As we have omitted signals extracted from the shape of the ROI, which require models specifically trained for the task, we might suggest that the shape of the ROI is particularly important when using RGB images, which is difficult to observe when the patient is covered or in darkness. In general, the larger, ellipse-based measurement intervals provide between signals than small rectangles or selected measurement points. However, selecting signals based on their motion allows out-of-phase signals to be avoided and produces better waveform estimates. Examples of such signals are shown in the Appendix (Section 7). We have relied on the torso detection to track patient movement between time windows. Feature tracking, as used elsewhere,^41^ may improve tracking within windows and reduce artefacts introduced by small movements.

While camera-based respiratory rate measurement is technically interesting, we should consider the clinical benefits of the system. The camera system does not require any contact-based sensors. The valid measurement time when either camera or patient monitor is much higher than when just one is available (75.3% vs 57.9%), reducing the time without valid measurements. In addition, when both measurements are valid (40.4%), the overall mean absolute error between the two is under 2 breaths/minute using either evaluation metric, indicating that measurements are reliable.

The processing pipeline requires modest computation capacity; the most significant part is the pose estimation which is suitable for 3 frames per second inference on consumer laptop (Apple MacBook Air, 2022), or around 90 frames per second using a modest consumer GPU (NVIDIA RTX 3060Ti). The latter would support computation for an entire NICU room. In both cases, video data would be kept on-site and not be retained beyond the initial signal extraction process. While the potential for recording sensitive video may be a concern for parents, data management procedures are already in place for video-EEG where data is intentionally retained. There is likely no requirement to use our specific RGB-D camera. We downsample the images to 256 × 256 for pose estimation, and the depth grid is 12 × 12, so a range of hardware may be suitable provided it incorporates the required sensors and is not affected by recording into the incubator. Research on varying the image resolution in adult respiratory monitoring with RGB cameras has been inconclusive, where Massaroni et al.^42^ find that reducing the image size decreases performance, but Zhang et al.^41^ reduce the image size to 320 × 240 without performance loss.

Beyond the aforementioned lack of gold standard apnoea detection, we should also examine the relative size of each subset within the data. We note that there are few patients from minority ethnic backgrounds, though for those included, the camera method appears to be more accurate. Given the use of depth cameras, in which skin colour is not visible, we would not expect a difference in performance, but this should not be assumed. All participants were in incubators, who are typically smaller than those elsewhere in the NICU, and at greatest risk of skin damage from contacting wires and are at the greatest risk of respiratory disorders. The incubator also provides a constant imaging environment, potentially mitigating differences between hospitals. Demonstrating applicability in the entire NICU–including term babies–would require further data collection in multiple sites, and a particular focus on smaller (<1000 g) patients, those with CPAP respiratory support, and including a cohort of increased ethnic diversity to confirm whether these factors affect measurement accuracy.

The methods used to assess confidence intervals has some potential limitations; for a given participant, we estimate the distribution by bootstrapping blocks, rather than the individual samples, effectively describing a 24 h recording as (for example) 48 30-min segments, which are sampled from the overall recording. This ensures that the samples retain the expected inter-sample correlation. There are small gaps within the intervals, due to invalid data, SQIs, or subset division, particularly when dividing the dataset by respiratory rate, which is not accounted for. There is no belief that this method is biased in any direction, but a detailed theoretical analysis of the statistical methods would be necessary.

In summary, we have collected a dataset of 437 h of RGB-D camera and patient monitor data, recorded over 24-h periods in neonatal intensive care–the longest of its kind, particularly regarding the length of each individual recording. Our respiratory rate measurement method is fully automated, with errors under 2 breaths/minute during valid intervals. Recordings represent a complete day of respiratory rate measurement, regardless of the patient's environment and behaviour. This work is the first to undertake this evaluation and demonstrate potential feasibility of continuous respiratory rate measurement using the camera in the NICU.

The camera system achieves 60% valid measurement time with MAE under 2 breaths/minute difference from the patient monitor when averaged across all data. Both the patient monitor and camera have reduced validity during periods of low respiratory rate, and the combined valid measurement availability is increased from around 58%–75% with a useful double-check provided 40% of the time. This can improve hospital e-records by recording samples when both camera and patient monitor agree. In addition, integrating a camera system into NICU care has potential to offer richer respiratory data capture in the future, such as paradoxical breathing, elevated respiratory effort or distress, and the patient's tolerance to changes in respiratory support, as well as additional monitoring such as seizures, sleep or movement quality. We have not specifically considered detection of alarm trigger levels or apnoeas; we would suggest that a future study in this area should include additional measurement methods, such as capsule pneumography, to provide the necessary ground truth.

## Contributors

JL & KB jointly led the conceptualisation of the study, secured funding and supervised the project. AG was responsible for data curation and analysis, investigation, software, and visualisation. LT provided study resources and also conducted the investigation. AG wrote the original draft; JL and KB contributed to the review and editing. All authors had access to the study data and the decision to submit. AG and KB accessed and verified the underlying data.

## Data sharing statement

We are unable to share the video recordings due to patient privacy. We are able to share the patient monitor data and extracted signals upon reasonable request to the authors. Code to implement the processing pipeline will be available from www.github.com/ajgrafton/neonatal-rr-monitoring.

## Declaration of interests

KB and JL are recipients of an NIHR i4i Product Development Award for development of a Bluetooth vital sign monitoring system. KB also holds shares in a related patent, has received funding from Diabetes UK, is East of England Paediatric Speciality Lead for RDN, and is NIHR HRC theme lead for MedTech collaboration. The other authors declare no competing interests.
