# Supplementary material for: Camera-based respiratory rate monitoring in unmodified neonatal intensive care – a cross-sectional study in a UK hospital
Source: eClinicalMedicine. 2026 Aug 31;99:104175. doi: 10.1016/j.eclinm.2026.104175 (PMC13562425; doi:10.1016/j.eclinm.2026.104175)
Supplement: Supplementary file 1 [file mmc1.pdf]

Camera-Based Respiratory Rate Monitoring in Unmodified Neonatal Intensive Care - A Cross-Sectional Study in a UK Hospital

Appendix

Contents

1 Camera-Monitor Time Synchronisation **2**

2 Spectral Clustering **4**

3 Measurement Error Arising from Window Parameters **5**

4 Patient Monitor Signal Processing **6**

5 Linear Sum Assignment **7**

6 Signal Quality Index (SQI) Implementation **8**

7 Comparison with Existing Work **10**

8 Dataset **12**

9 Additional Results **13**

10 ECG Waveform Analysis **20**

11 Sample Size Calculation **22**

1 Camera-Monitor Time Synchronisation

This section describes the process for aligning camera and patient monitor data.

Camera frames are timestamped by the camera using its own internal timestamp (which is not aligned with any external clock) and the time it was received by the computer. We initialise the recording using the computer time of the first frame, and subsequent sample timestamps are based on the difference between the camera's internal timestamp for successive frames. This eliminates the effect of any computer-side processing delay. The patient monitor sends blocks of waveform samples over IP. They are timestamped according to the time that they are first received, with each sample within the block offset by the patient monitor's sample period (1/60s), and subsequent blocks appended with the same sample period. We expect low latency between the monitor and the computer due to the small data volume and direct ethernet connection; the camera is more likely to drop frames.

We find that there is a delay between the camera and patient monitor; usually up to 20 seconds. The camera signals are aligned to the patient monitor as follows:

1. View the patient monitor and camera signals side-by-side and identify obvious points where the signals match; this is often achieved by finding periods of movement to understand the approximate offset, then finding particularly long or short breaths to accurately align the signals.

2. Alignment points are chosen approximately every 15 minutes. Where either recording has a pause, additional alignment samples are inserted just after or just before the pause, as the offset may be different when recording resumes.

3. To correct a camera time, use a piecewise linear interpolation of the camera alignment sample times and the offset (monitor time minus camera time). Add the interpolated offset onto the camera time.

All signal processing (breath calculation, signal quality indices) is done before the alignment. Comparison is done in monitor time to (a) avoid affecting the patient monitor signal and (b) the monitor data likely has less latency. Figure 2 illustrates some alignment samples. Example signals from excluded patients are provided in Figure 1, where the required visible features are missing.

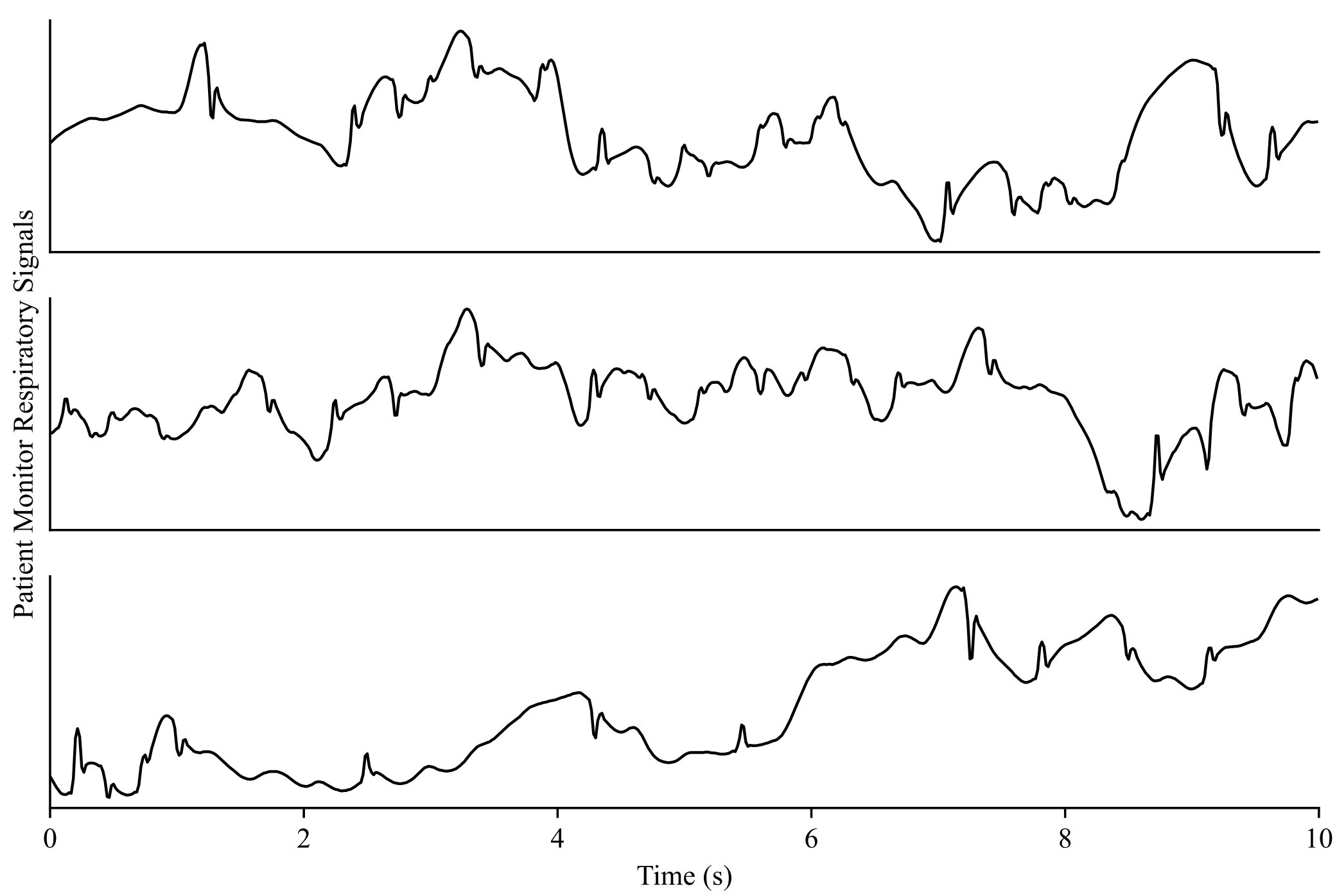

Figure 1: Examples of respiratory signals from excluded participants. The vast majority of the data from each of these participants was similar to the examples, making correct synchronisation impossible.

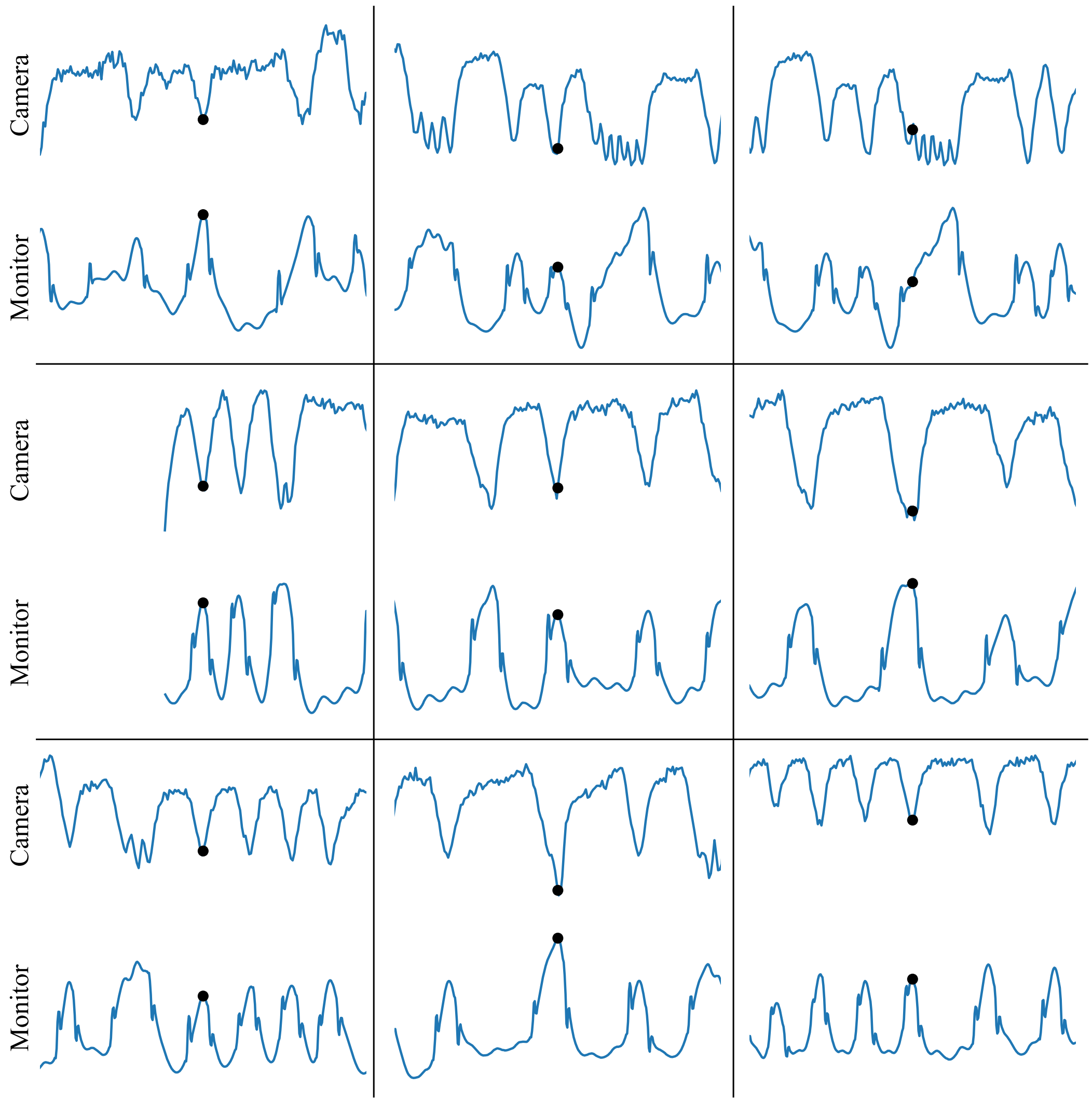

Figure 2: Examples of time synchronisation samples. Nine examples are shown, used to synchronise a one-hour period. The centre-left sample is used to restart the alignment just after a recording gap. The marks show the selected point from the camera and monitor signal. Camera troughs typically correspond to monitor peaks.

2 Spectral Clustering

We use a spectral clustering method,[1] with the QR clustering approach[2] as it provides deterministic solutions. This method requires similarity scores instead of considering each 20s window as a 600-dimensional value. For a signal at grid square (i, j), denoted z_{ij}, we first subtract its mean and calculate the discrete Fourier transform, Z_{ij}. When comparing signals, we would like two pure noise signals to have high similarity, so coefficients corresponding to > 100 breaths per minute are replaced by their absolute value. The rationale is that for low frequencies (representing movement) and medium frequencies (representing respiration) we do care about the signal's phase, since two out-of-phase signals would destructively interfere when averaged, but for high frequencies (noise), we simply wish to quantify the amplitude. The correlation coefficient between the two signals at grid squares (i_1, j_1) and (i_2, j_2) is given by

$$C_{(i_1,j_1),(i_2,j_2)} = \frac{Z_{i_1,j_1} \cdot Z_{i_2,j_2}}{\sqrt{\left(Z_{i_1,j_1} \cdot Z_{i_1,j_1}\right)\left(Z_{i_2,j_2} \cdot Z_{i_2,j_2}\right)}} \tag{1}$$

Where each dot product takes the complex conjugate of the second operand such that C is real. We add 1 to ensure positive C, followed by a spatial similarity to get nearby signals high affinity, where $s = 3$ weights the value and $\epsilon = 0.3$ ensures non-zero denominators, giving the affinity matrix A, used in the spectral clustering algorithm.

$$A_{(i_1,j_1),(i_2,j_2)} = C_{(i_1,j_1),(i_2,j_2)} + 1 + \frac{s}{(i_2 - i_1)^2 + (j_2 - j_1)^2 + \epsilon} \tag{2}$$

3 Measurement Error Arising from Window Parameters

This section provides further examples of how a measurement error in the respiratory rate may appear due to differences in the window position or window time for calculation. We choose data from participant 9 (the highest camera valid time) and calculate the respiratory rate using a range of window times (6-14s). The rate is calculated every two seconds; we also offset the measurements by 0·5s and 1·0s and interpolate in between. This represents an identical measurement method, but with the window times slightly shifted. The calculated MAEs are listed in Table 1.

The MAE further increases if we add a small time delay between measurements. Table 2 and Table 3 show the errors if the signals are shifted by two and five seconds respectively, representing a time synchronisation error. This could also represent a delay in the patient monitor's respiratory rate measurement output, compared to its waveform output.

These results are produced using the same breath positions; only the rate calculation method is changed. Given that their amplitude is similar to the MAE values reported in the main text, this shows that patient monitor data should be re-processed using a known method, and that careful time-synchronisation is needed. The errors presented here are from the neonatal population, and are likely worse than would be found in adults, due to neonates' highly variable respiratory rate.

Table 1: Mean absolute error (MAE) introduced by varying the sliding window length for respiratory rate calculation. The respiratory rate is calculated twice, using the row- and column-heading window lengths. The offset refers to a time shift in the window position, which is interpolated across to find the respiratory rate. Each entry in the table is the MAE calculated when the same breaths are processed using the two window lengths. Values are given in breaths per minute.

Window	No Offset					0·5s Offset					1·0s Offset				
Length (s)	6	8	10	12	14	6	8	10	12	14	6	8	10	12	14
6	0·00	1·38	2·56	2·90	3·61	0·68	1·45	2·34	2·97	3·43	0·92	1·60	2·26	3·17	3·37
8	-	0·00	1·15	2·14	2·49	-	0·51	1·21	1·97	2·54	-	0·69	1·33	1·91	2·68
10	-	-	0·00	0·98	1·83	-	-	0·39	1·01	1·71	-	-	0·53	1·10	1·66
12	-	-	-	0·00	0·87	-	-	-	0·34	0·90	-	-	-	0·45	0·96
14	-	-	-	-	0·00	-	-	-	-	0·30	-	-	-	-	0·40

Table 2: Mean absolute error (MAE) introduced by varying the sliding window length for respiratory rate calculation. A two-second time synchronisation error is also added.

Window	No Offset					0·5s Offset					1·0s Offset				
Length (s)	6	8	10	12	14	6	8	10	12	14	6	8	10	12	14
6	3·88	3·29	3·39	3·40	3·88	3·68	3·35	3·28	3·45	3·71	3·61	3·50	3·26	3·59	3·66
8	-	3·10	2·71	2·87	2·95	-	2·95	2·75	2·80	2·99	-	2·89	2·85	2·78	3·09
10	-	-	2·55	2·33	2·49	-	-	2·45	2·36	2·44	-	-	2·41	2·45	2·43
12	-	-	-	2·22	2·03	-	-	-	2·13	2·06	-	-	-	2·10	2·13
14	-	-	-	-	1·93	-	-	-	-	1·86	-	-	-	-	1·83

Table 3: Mean absolute error (MAE) introduced by varying the sliding window length for respiratory rate calculation. A five-second time synchronisation error is also added.

Window	No Offset					0·5s Offset					1·0s Offset				
Length (s)	6	8	10	12	14	6	8	10	12	14	6	8	10	12	14
6	7·30	6·86	6·07	5·76	5·24	7·37	6·70	6·11	5·65	5·27	7·58	6·65	6·25	5·61	5·36
8	-	6·10	5·72	5·16	4·90	-	6·15	5·62	5·19	4·81	-	6·27	5·58	5·29	4·79
10	-	-	5·15	4·90	4·44	-	-	5·18	4·81	4·47	-	-	5·28	4·78	4·55
12	-	-	-	4·45	4·24	-	-	-	4·48	4·16	-	-	-	4·57	4·14
14	-	-	-	-	3·88	-	-	-	-	3·91	-	-	-	-	3·97

4 Patient Monitor Signal Processing

This section describes the signal processing method for identifying breaths in the patient monitor waveform. Each breath detection is characterised by a notch on the waveform, shown in Figure 3. We use a matched filter with coefficients 2, -4, -20, -20, -4, 2, adjusted to zero mean and unit norm, and locate positive/negative peaks in the filtered signal whose magnitude exceeds a threshold. Peaks must also be the largest absolute value up to three samples in each direction (the matched filter causes small negative peaks to appear either side of a positive peak, and vice-versa). Peaks are then removed to ensure an alternating sequence of positive/negative peaks; if there are multiple consecutive positive peaks, the largest is retained. Local maxima in the original waveform, the maximum between each pair of down- and up-notches, denote the start and end of breaths.

The threshold is found separately for each recording interval such that the valid fraction (based on the signal quality indices) is maximised, to ensure that we do not unfairly penalise the patient monitor by configuring the algorithm inappropriately.

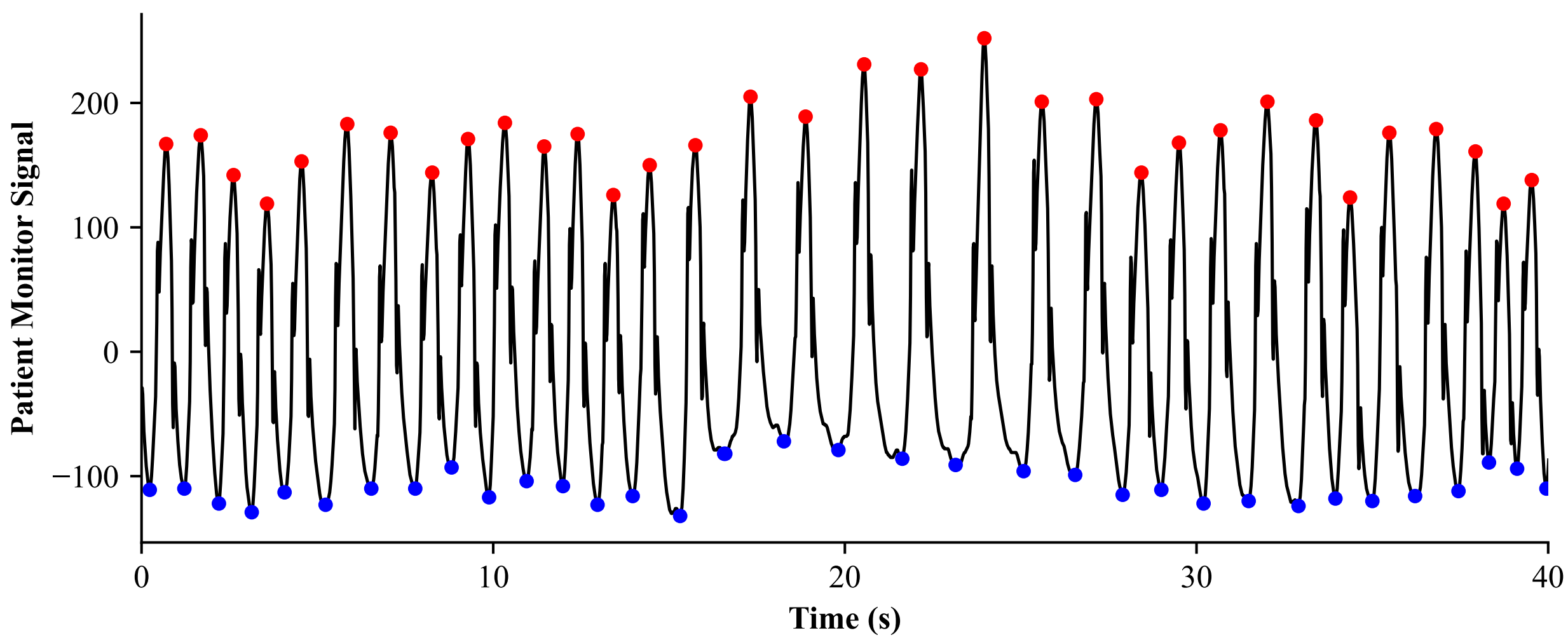

Figure 3: Example patient monitor data. Inhalation and exhalation are marked by the patient monitor as a notch on the signal. Red and blue dots indicate the detected peaks and troughs, respectively.

5 Linear Sum Assignment

This section describes the method of comparing the camera and patient monitor breaths as a linear sum assignment problem.

We aim to match breaths found by the camera and patient monitor in order to find missing or extra breaths, which would contribute to an error in the respiratory rate measurement. The linear sum assignment method, given a matrix A, aims to maximise:

$$\sum_{k=1}^{K} A_{c_k, m_k} \tag{3}$$

where $\{c_k, m_k\}$ are the sequence of matched camera and monitor breaths, respectively, and K is the total number of matches. Each camera and monitor breath may appear only once in $\{c_k, m_k\}$. We design the matrix A, with dimensions (number of camera breaths $\times$ number of monitor breaths). The solution provides the sequence of assignments. If a camera breath is not assigned, it is an 'extra' breath, and an unassigned monitor breath is a 'missing' breath.

Elements of A as given as follows:

$$A_{ij} = \begin{cases} 1 + \mathrm{IoU}(i, j) \times 10^{-6} & \mathrm{IoU}(i, j) > 0 \\ 0 & \text{otherwise} \end{cases} \tag{4}$$

where $\mathrm{IoU}(i, j)$ is the intersection-over-union between camera breath i and monitor breath j. The 10^{-6} factor ensures that matching the maximum number of breaths is prioritised over the total IoU. However, without the IoU in the calculation, the solution may not be unique. Maximising the number of breaths plus the IoU gives the most sensible collection of matched breaths. We need the missing/extra breaths to be where we would expect them to be, otherwise they will not coincide with poor signal quality.

6 Signal Quality Index (SQI) Implementation

This section describes the implementation details of the signal quality indices.

To calculate the Peak-Trough Amplitude SQI, we compute the height difference (in millimetres for depth, or the patient monitor waveform units), between each peak-trough or trough-peak pair. If a peak-trough absolute height difference is greater than 3x the preceding trough-peak absolute height difference, we label the peak-trough interval, as well as preceding and following trough-peak intervals, as invalid. The same is applied to each trough-peak interval, based on the absolute height difference of the preceding peak-trough interval. This is illustrated in Figure 4.

To calculate the Shape SQI, we examine each breath (trough-trough for the camera, or peak-peak for the patient monitor) individually. For a perfect breath shape, we expect that the gradient is all positive and then all negative, or vice-versa. We calculate the sum of absolute differences between sample points (total difference). For the camera, we calculate the absolute difference between the peak and first point plus the absolute difference between the peak and the last point; for the patient monitor, we use the trough. This gives the 'perfect difference.' For sample points $x_{1:n}$, this is given by:

$$\text{Total Difference (TD)} = \sum_{i=1}^{N-1} |x_{i+1} - x_i| \tag{5}$$

$$\text{Camera Perfect Difference (PD)} = 2\max(x) - x_1 - x_N \tag{6}$$

$$\text{Monitor Perfect Difference (PD)} = x_1 + x_N - 2\min(x) \tag{7}$$

If the value $\frac{TD-PD}{PD}$ exceeds a threshold, the SQI is set to 1, and is zero otherwise. We also check each consecutive three breaths; if the three breaths give an average respiratory rate exceeding 120bpm, the SQI is set to 1 over the three breaths. This is needed to prevent the patient monitor selection threshold from choosing as many breaths as possible, and also rejects times when the patient monitor is incorrectly recording the patient's heart rate. The calculation is illustrated in Figure 5.

Before applying this to the patient monitor signal, we remove the detected notches by replacing the five samples, centred on the notch, with a quadratic fit of the two points to the left and two points to the right of the interpolated samples. The notches would otherwise count against the signal quality.

Examples where each SQI is triggered are shown in Figure 6.

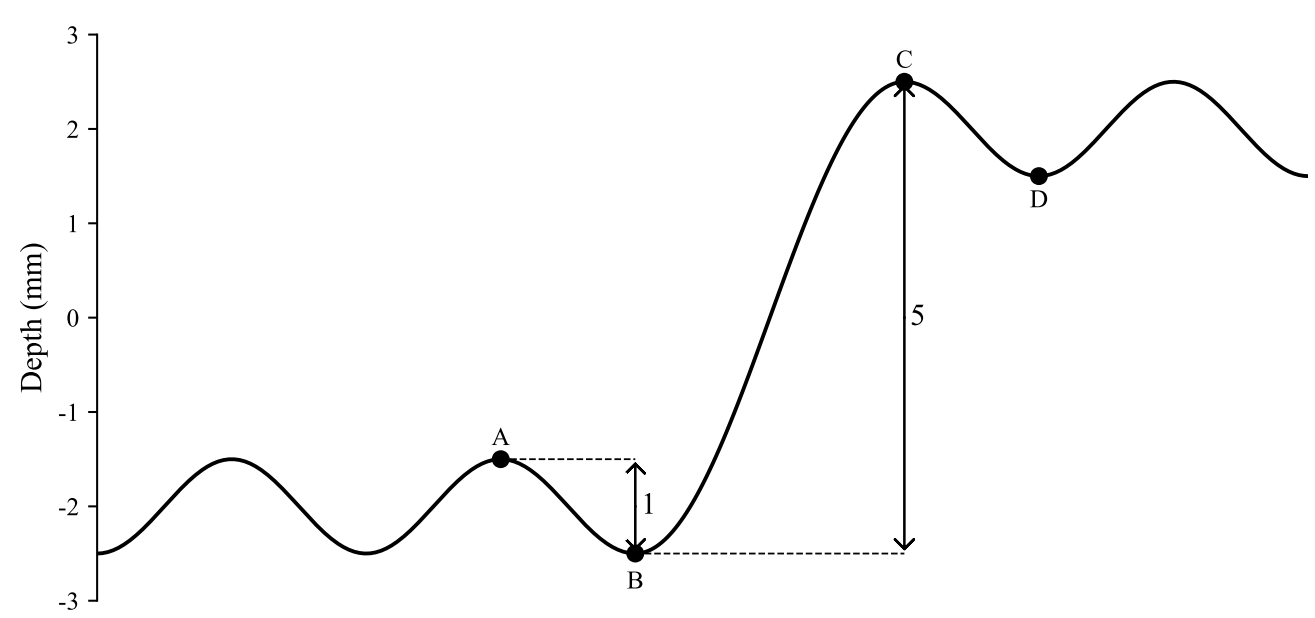

Figure 4: Illustration of the Peak-Trough SQI. The height difference between points B and C is 5mm, which exceeds three times the height difference between points A and B (1mm). As such, the interval B-C is labelled as invalid, as are the preceding (A-B) and following (C-D) peak-trough intervals.

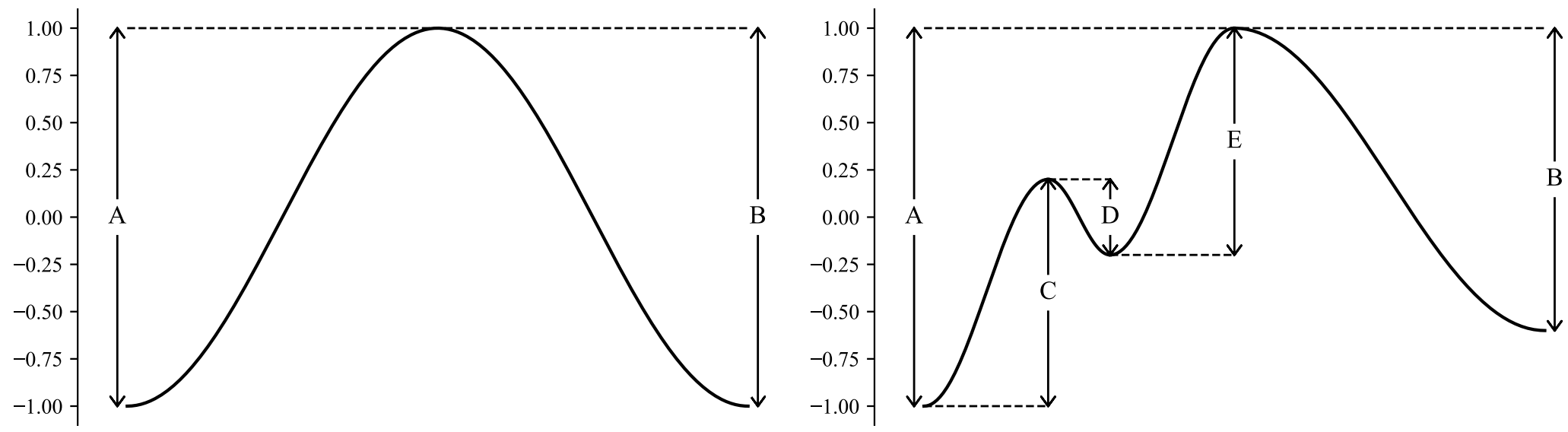

Figure 5: Illustration of the Shape SQI. In the left-hand figure, the perfect difference and total difference are both $A + B$; the SQI threshold will never be exceeded. In the right-hand figure, the perfect difference is $A + B$, which is 3.6 in this example. The total difference is $C + D + E + B = 4.4$, giving a value of $(4.4 - 3.6)/3.6 = 0.22$.

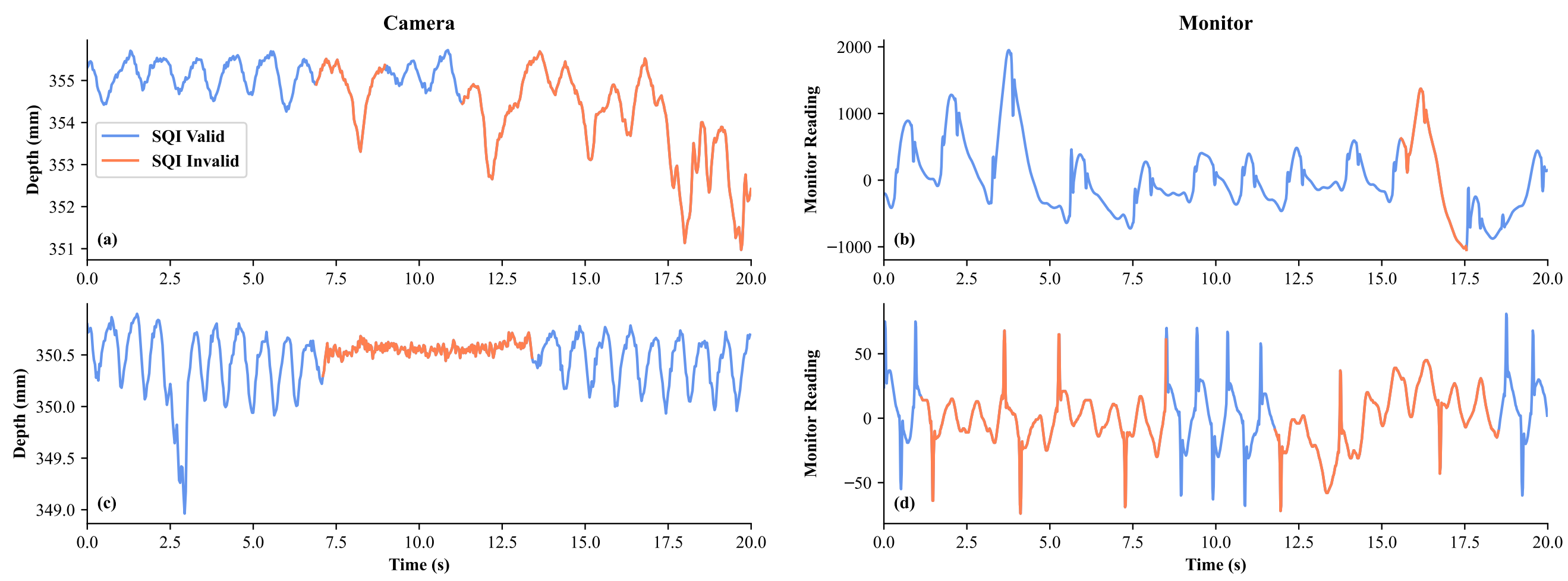

Figure 6: Examples where each SQI is triggered in camera and patient monitor data. a, b: Peak-trough amplitude SQI examples for camera and patient monitor; **c, d**: Shape SQI examples for camera and patient monitor. The shape SQI for the monitor triggers because there are multiple oscillations without notches, coinciding with a decrease in measured respiratory rate, indicating that the patient monitor is failing to detect these breaths.

7 Comparison with Existing Work

This section describes the modifications made to each existing method to adapt to our dataset.

Villarroel et al. (2019).[3] This work uses an automatic approach for selecting skin regions; a region of interest (ROI) for extraction is found by fitting an ellipse to the region. Twelve signals are used to estimate respiratory rate: three from the average RGB values within the ellipse, and the remainder from the parameters of the chosen ROI. Peak finding on each signal is used to calculate the respiratory rate. The method is modified as follows:

- We manually annotate skin areas for each window (every second) rather than every frame, which would be infeasible. The ellipse is found using Welzl's method,[4,5] and the ellipse parameters are not used in the respiratory rate extraction.
- We use five colour channels (RGB, depth, infra-red) instead of just RGB.
- The moving average curve (MAC) peak finding approach[6,7] is modified by using three-second windows for the moving average. The range of the signal is found for each three-second window, and up/down thresholds are set 55% and 45% respectively to prevent repeated crossings due to noise.
- The boxed slope sum function (BSSF)[8] is modified by adding a decay to the peak threshold, decaying from 0.7 to 0.5 of the peak amplitude to prevent motion from causing large peaks and setting thresholds too high.
- The filter is implemented using a 15-pole 300 breaths per minute low-pass Butterworth filter. This, and the above peak finding adjustments, improve performance on our dataset.

The peaks are used in either of our evaluation methods.

Cenci et al. (2015).[9] This work uses depth data, by averaging the depth at manually selected measurement points. We replicate this by calculating seven measurement points from the ellipses found previously: one in the centre, and six equally spaced by angle around the centre, half-way between the centre and perimeter. Although the original work does not adjust measurement points over time, we do so as our recordings are longer and the baby is likely to move. The depth is taken as the average of the 3x3 area centred on the measurement point, to account for possible invalid depth pixels. A fourth-order Butterworth filter is used to implement the smoothing. Peaks are found as local maxima. The original work calculated an instantaneous respiratory rate using the time between consecutive peaks. As we have identified breaths in the signal, we instead use these peaks with our evaluation metrics.

Cobos-Torres et al. (2018).[10] This work uses grayscale signals from a manually selected small skin area. We use a skin area with similar size to the original work, with the same centre as the previous ellipses. As the authors' method requires annotations every 3/8 seconds, we interpolate the ellipse centres over time and manually verify the results. We use the same time-windowing approach as in our signal processing to allow the skin area to move during the recording. The method is applied using grayscale, depth and infra-red images. The original work used a 30-60 breaths per minute filter, which we implement using a fourth order Butterworth filter. Due to the narrow passband of the filter, which corresponds to a normal neonatal respiratory rate, we also investigate a 20-100 breaths per minute filter, implemented using an eighth order Butterworth filter. The respiratory rate is calculated using a short-time Fourier transform with step size 0.1 seconds. For evaluation, we average the rate over two-second windows (the patient monitor sample spacing) and use the rate comparison evaluation method. The breath matching method is not used as this approach uses spectrum analysis rather than breath finding.

We present some examples showing the utility of our signal processing and extraction. Figure 7 compares the signal extracted from the ellipse with our clustering. We typically find that our signal extraction approach avoids averaging out-of-phase signals, leading to a higher measured amplitude. We also compare our extraction and signal processing with measurement point signal extraction in Figure 8. By finding blocks of continuous positive or negative gradient, we are able to avoid noise without using a filter which may introduce ringing artefacts leading to spurious detections.

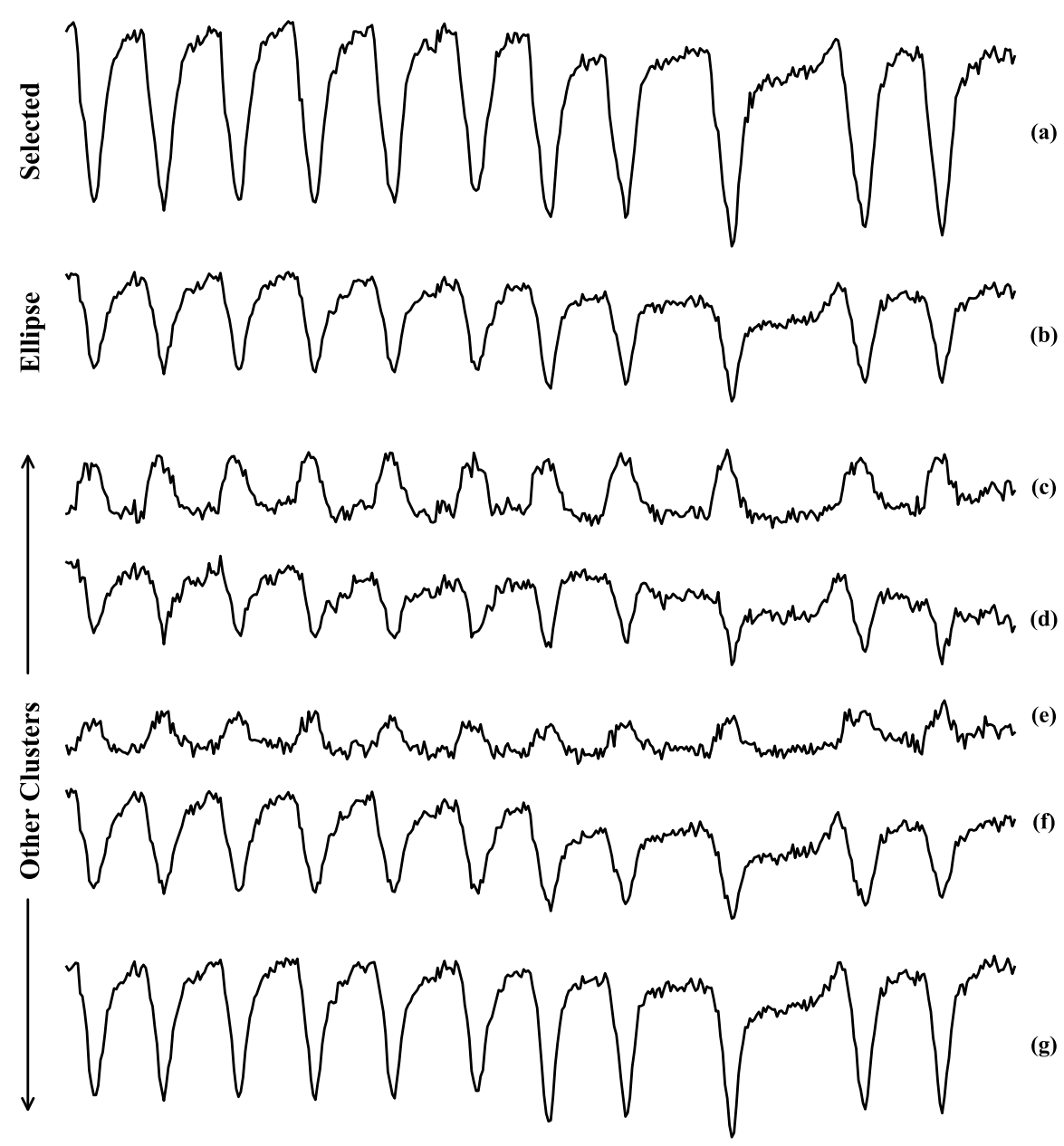

Figure 7: Comparison of our cluster-extracted depth signals and the average depth over an ellipse. a: Our chosen extracted signal, **b**: Signal extracted from the average ellipse depth, **c**-**g**: other cluster signals, not selected. Each signal is shown at the same scale. Note that signals in **c** and **e** are out of phase with the selected signal, and would reduce its amplitude if included in the averaging.

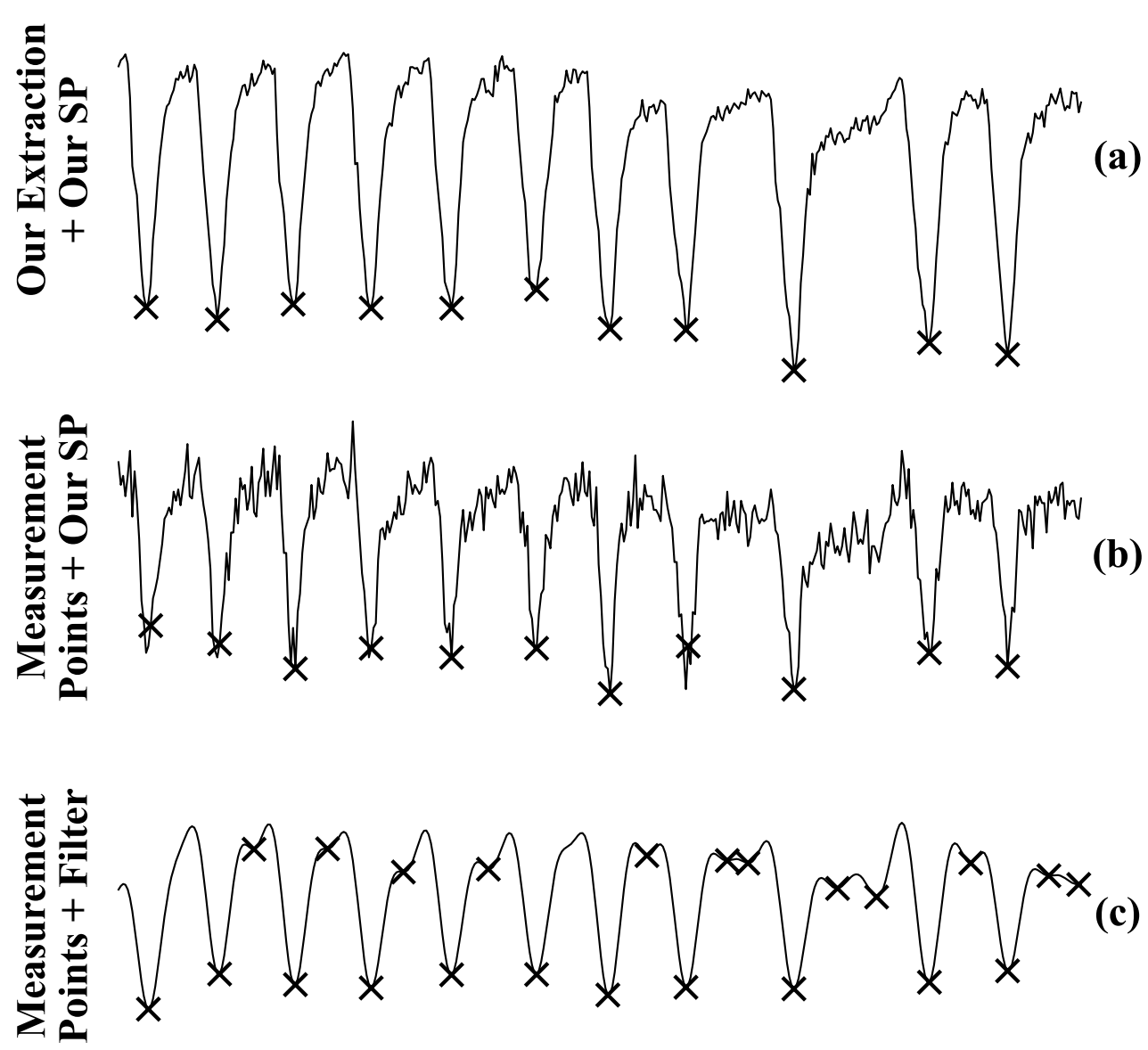

Figure 8: Comparison of measurement point plus filtering/peak-finding with our extraction and processing method. a: Our selected signal with troughs highlighted using our signal processing method. **b**: The same time period with measurement point signal extraction and our signal processing; the signal is noisier, but the same breaths are found. **c**: The same measurement point signal, filtered and troughs used; the filtering produces oscillations, particularly in breaths with a plateau rather than a peak. Each signal is shown at the same scale.

8 Dataset

Histograms of participants' respiratory rates are presented in Figure 9, divided by intervention/non-intervention, activity and covering, and further by the monitor SQI validity. Including only measurements where the patient monitor waveform is considered valid reduces the mean, median and quartile values, partly due to a concentration of values at around 170 bpm, occurring when the monitor measures heart rate instead of respiratory rate, and is removed by the SQI calculation. This occurs for three participants only.

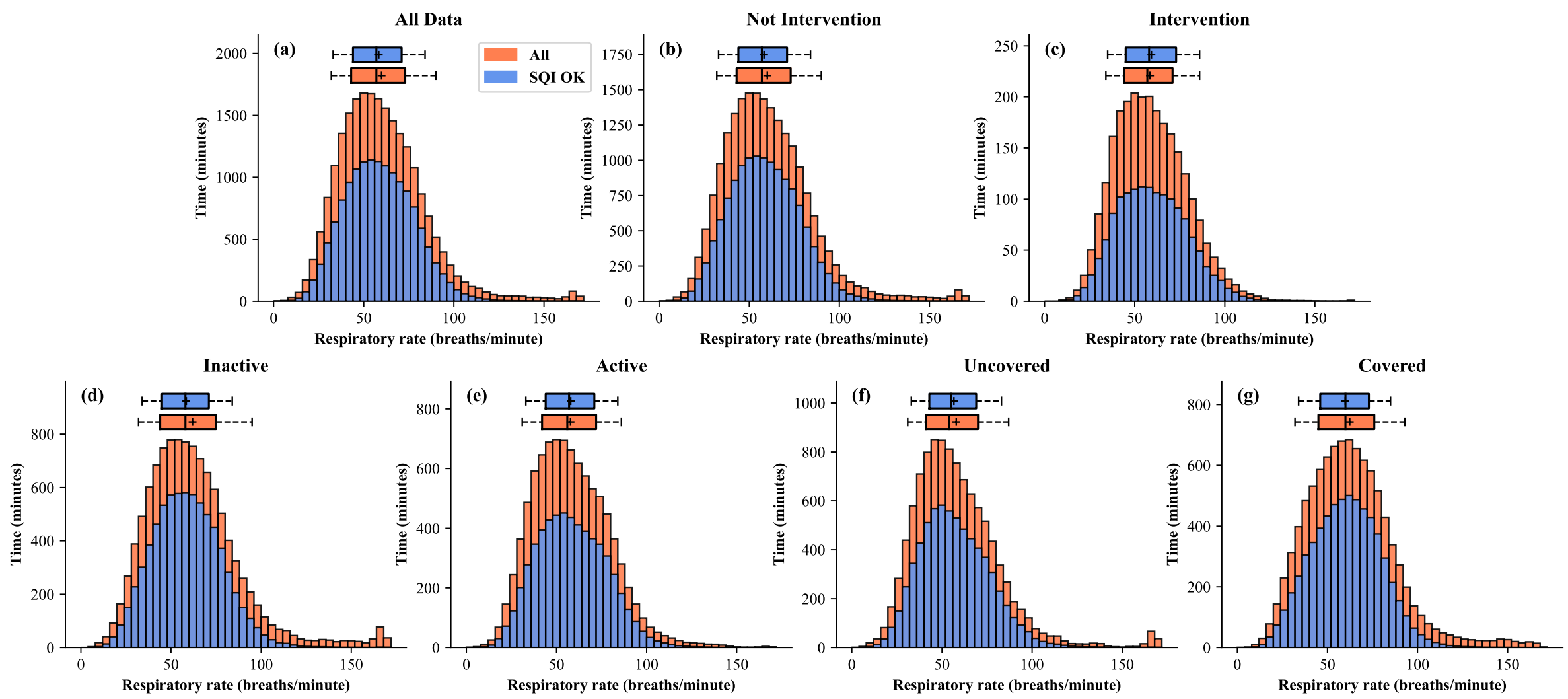

Figure 9: Histograms of respiratory rate for study participants. The dataset is divided by activity, covering and intervention: **a**: all data, **b**: non-intervention data, **c**: intervention, **d**: inactive, **e**: active, **f**: uncovered and **g**: covered. The red histogram 'All' includes all data, and the blue histogram 'SQI OK' only shows samples where the patient monitor SQI is valid.

9 Additional Results

The mean absolute error and bias for the PCA-based extraction method are presented in Table 4 alongside the same results for our clustering method, which are repeated from the main text. The recording times for each participant in the dataset are summarised in Table 5, and the valid measurement time, mean absolute error and bias are summarised in Table 6. The data is divided by the patient's ethnicity, respiratory support, and other demographic information, with results presented in Table 7. Table 8 shows the results divided by the patient monitor's respiratory rate, and Table 10 shows the results divided by day shift (8am-8pm) and night shift. The results are repeated for varying measurement window size in Table 11. There is naturally a trend that increasing the window size improves the measurements as consecutive missed/extra breaths cancel out. The significance markers indicate significance of the result if an additional 20 participants were recruited and the measurements repeated with this window size - would it be obvious that a different window size has been used - in the Table, they draw attention to values that have been notably modified by the change in window size. Bland-Altman plots for the overall dataset are given in Figure 10.

Table 4: Mean absolute error (MAE) and bias for valid measurement intervals. Positive bias indicates that the camera is measuring a higher rate than the patient monitor. Only time periods where both camera and patient monitor are valid are shown. MAE and bias values are given in breaths/minute. Error is calculated using breath-by-breath matching and measured respiratory rate comparison. Signals are extracted using the spectral clustering method (these results mirror the main text), and a PCA method where candidate signals are found using PCA and selected using the same spectral content method. The percentages of the data used for measurement are also shown; for the rate comparison method, the 'valid' column lists the percentage of time that measurement is available if one sample is required every 30 seconds. The clustering method outperforms the PCA method in almost all metrics and evaluation methods.

	Clustering Method						PCA Method					
	Breath Matching			Rate Comparison			Breath Matching			Rate Comparison		
Subset	MAE	Bias	Valid	MAE	Bias	Valid	MAE	Bias	Valid	MAE	Bias	Valid
All	1·52	0·46	39%	1·58	0·38	27%	1·58	0·89	33%	1·61	0·59	22%
Intervention	2·43	0·86	28%	2·66	0·85	15%	2·43	1·09	23%	2·70	0·84	11%
Not Intervention	1·43	0·43	40%	1·51	0·35	28%	1·51	0·87	35%	1·54	0·58	23%
Uncovered	1·47	0·59	45%	1·43	0·59	33%	1·53	0·94	41%	1·44	0·76	28%
Covered	1·39	0·21	36%	1·62	-0·02	24%	1·48	0·76	28%	1·72	0·25	17%
Inactive	1·19	0·26	43%	1·28	0·19	33%	1·25	0·68	38%	1·35	0·39	28%
Active	1·77	0·67	37%	1·90	0·62	23%	1·89	1·15	31%	1·88	0·92	18%
Uncovered, Inactive	1·18	0·31	50%	1·11	0·37	40%	1·28	0·69	47%	1·20	0·57	36%
Uncovered, Active	1·87	0·97	39%	2·01	0·98	24%	1·89	1·30	35%	1·88	1·12	21%
Covered, Inactive	1·20	0·17	37%	1·54	-0·11	26%	1·20	0·66	30%	1·63	0·06	20%
Covered, Active	1·64	0·27	34%	1·75	0·13	21%	1·89	0·90	26%	1·87	0·57	15%

Table 5: Recording time for each participant. Unc=Uncovered, Cov=Covered, Ina=Inactive, Act=Active. All values are given in hours.

Number	Total Length	Intervention	Non-Intervention	Unc./Ina.	Unc./Act.	Cov./Ina.	Cov./Act.
1	23·3	2·5	19·8	6·7	2·2	7·0	3·9
2	23·7	2·4	18·0	12·5	5·4	0·0	0·0
3	24·0	1·4	22·2	8·1	2·4	3·6	8·1
4	24·1	1·6	16·8	3·2	4·6	5·1	3·9
5	22·9	1·6	19·9	3·2	3·1	8·5	5·2
6	24·5	2·6	21·9	0·0	0·0	10·2	11·7
7	24·1	0·0	24·1	8·4	15·7	0·0	0·0
8	22·9	1·5	14·3	4·2	1·1	5·9	3·1
9	11·0	0·1	7·7	0·3	0·3	5·9	1·2
10	14·2	0·7	10·8	1·6	0·0	3·3	5·8
11	9·4	0·1	5·8	4·9	1·0	0·0	0·0
12	21·8	2·8	14·5	5·4	1·7	4·4	3·0
13	23·2	3·1	19·8	1·1	1·1	15·5	2·2
14	23·7	2·4	19·0	5·2	3·5	9·6	0·7
15	23·8	1·4	19·0	2·8	4·5	5·3	6·5
16	23·9	1·2	18·3	4·5	12·6	0·0	1·2
17	23·5	4·0	19·5	6·0	13·5	0·0	0·0
18	23·0	3·6	16·9	0·0	0·9	3·9	12·1
19	24·7	4·3	16·1	12·1	4·0	0·0	0·0
20	23·0	5·7	12·9	2·4	7·4	1·2	2·0
Total	434·6	43·2	337·5	92·5	84·7	89·4	70·8

Table 6: Valid time and error for each participant during non-intervention periods. Length refers to the length of non-intervention recording. Time values are presented in hours and as a percentage of the overall length. The mean is averaged across each participant; the weighted means are weighted according to the length of the recording, and are the same as the results in the main text. MAE and bias are reported in breaths/minute. Eth. = ethnicity, reported as white (W) and minority ethnic background (excluding white minorities) (M). Supp. = respiratory support, reported as SV (self-ventilating), HFNC (high-flow nasal cannula), CPAP (continuous positive airway pressure) or Vent (ventilation).

Number	Length	Monitor Valid	Camera Valid	Either Valid	Both Valid	MAE	Bias	Eth.	Supp.
1	19·8	13·5 (68·0)	14·8 (74·9)	16·8 (84·7)	11·5 (58·2)	0·87	0·29	M	HFNC
2	18·0	14·4 (80·1)	13·6 (75·5)	15·6 (86·7)	12·4 (68·9)	0·77	0·39	M	HFNC
3	22·2	7·1 (31·7)	11·2 (50·4)	13·6 (60·9)	4·7 (21·1)	1·59	-1·14	M	CPAP
4	16·8	12·6 (75·2)	8·5 (50·5)	13·9 (82·6)	7·2 (43·1)	1·54	1·11	W	CPAP
5	19·9	13·0 (65·0)	8·6 (43·1)	14·6 (73·2)	7·0 (35·0)	1·14	-0·24	W	HFNC
6	21·9	9·3 (42·4)	10·7 (49·0)	15·0 (68·3)	5·1 (23·1)	1·18	-0·57	W	Vent
7	24·1	10·2 (42·3)	11·0 (45·7)	15·2 (63·1)	6·0 (24·9)	1·53	0·93	W	HFNC
8	14·3	8·4 (58·7)	8·1 (56·6)	10·6 (74·1)	5·9 (41·1)	1·55	0·65	W	CPAP
9	7·7	5·8 (75·3)	5·9 (77·4)	6·6 (86·4)	5·1 (66·3)	0·78	0·43	W	HFNC
10	10·8	6·2 (57·3)	6·0 (55·3)	7·8 (72·6)	4·3 (40·0)	1·92	0·06	M	HFNC
11	5·8	1·9 (32·0)	3·9 (67·2)	4·3 (73·6)	1·5 (25·7)	4·20	-3·89	W	HFNC
12	14·5	10·6 (73·4)	10·7 (73·9)	12·4 (85·3)	9·0 (62·0)	1·05	0·84	W	HFNC
13	19·8	12·4 (62·3)	4·8 (24·1)	13·5 (68·2)	3·6 (18·1)	1·24	-0·61	W	CPAP
14	19·0	12·5 (66·1)	11·5 (60·5)	14·9 (78·7)	9·1 (47·9)	1·09	-0·25	W	HFNC
15	19·0	12·7 (66·6)	14·4 (76·0)	16·4 (86·2)	10·7 (56·4)	1·50	0·86	W	CPAP
16	18·3	11·0 (60·0)	11·5 (62·9)	13·7 (75·2)	8·7 (47·8)	1·04	0·02	W	SV
17	19·5	10·5 (53·8)	13·3 (68·6)	15·2 (78·3)	8·6 (44·1)	3·04	2·27	W	CPAP
18	16·9	8·2 (48·2)	6·3 (37·2)	11·0 (65·0)	3·4 (20·4)	2·59	0·32	W	CPAP
19	16·1	8·8 (54·5)	11·0 (68·3)	12·6 (78·1)	7·2 (44·8)	0·85	0·55	W	HFNC
20	12·9	6·6 (50·8)	9·1 (70·4)	10·2 (79·2)	5·4 (42·0)	3·07	1·74	W	HFNC
Mean	16·9	9·8 (58·2)	9·8 (59·4)	12·7 (76·0)	6·8 (41·5)	1·63	0·19		
(Weighted)	-	10·3 (57·9)	10·3 (57·8)	13·5 (75·3)	7·1 (40·4)	1·53	0·28		

Table 7: Valid time and error for each participant during non-intervention periods, divided by ethnicity and respiratory support. Time values are presented in hours and as a percentage of the overall length. The 'All' subgroup mirrors the results in the main text and is presented for convenience. MAE (mean absolute error) and bias are reported in breaths/minute. Ethnicity is given as white or minority ethnic background excluding white minorities (MEB). Respiratory support is given as SV (self-ventilating), HFNC (high-flow nasal cannula), CPAP (continuous positive airway pressure) or Vent (ventilation). MAE is below 2 breaths/minute and bias is with ±1 breath/minute in all cases. 95% confidence intervals are given. Significant results are given in bold, where *, **, and *** indicate that the method performs worse (relative to all non-intervention data) on this subset (further from zero) at the p = 0.05, 0.005 and 0.0005 levels respectively, and †, ††, and ††† indicate that the method performs better on this subset (closer to zero) at the same levels.

Subset	N	Monitor Valid		Camera Valid		Either Valid		Both Valid		MAE		Bias	
All	20	57·9	9·8	57·8	9·8	75·3	12·7	40·4	6·8	1·43	(1·16 – 1·80)	0·43	(0·07 – 0·78)
White	16	57·9	9·6	56·1	9·3	75·1	12·5	38·8	6·5	1·55	(1·23 – 1·96)	0·54	(0·10 – 0·96)
MEB	4	58·0	10·3	64·4	11·4	75·9	13·4	46·5	8·2	**1·07**†††	(0·81 – 1·77)	**0·09**†	(-0·62 – 0·37)
SV	1	68·0	13·5	74·9	14·8	84·7	16·8	58·2	11·5	**0·87**†††	(0·79 – 0·94)	**0·29**†††	(0·21 – 0·37)
HFNC	11	61·3	9·4	62·9	9·7	77·7	11·9	46·5	7·1	1·24	(0·96 – 1·70)	0·35	(-0·02 – 0·70)
CPAP	7	55·8	10·2	51·8	9·5	73·2	13·5	34·4	6·3	**1·89***	(1·48 – 2·46)	**0·77***	(-0·10 – 1·46)
Vent	1	42·4	9·3	49·0	10·7	68·3	15·0	23·1	5·1	**1·18**†	(1·08 – 1·29)	-0·57	(-0·69 – -0·46)
Under 30 Weeks	9	52·0	9·5	58·1	10·6	73·6	13·5	36·5	6·7	1·63	(1·23 – 2·19)	0·50	(-0·28 – 1·16)
Over 30 Weeks	11	63·5	10·0	57·5	9·0	76·8	12·1	44·1	6·9	1·28	(0·99 – 1·74)	0·38	(0·09 – 0·68)
Under 1000g	5	46·5	8·4	56·2	10·2	69·8	12·6	32·9	6·0	**1·97****	(1·28 – 2·92)	0·39	(-1·18 – 1·59)
1000-1250g	7	57·3	9·5	58·5	9·7	75·1	12·4	40·8	6·7	1·42	(1·17 – 1·78)	0·41	(-0·03 – 0·73)
Over 1250g	8	66·3	10·9	58·2	9·5	79·2	13·0	45·4	7·4	1·18	(0·86 – 1·71)	**0·47**†	(0·11 – 0·87)
Male	11	64·2	9·3	61·8	9·0	79·4	11·5	46·6	6·8	1·54	(1·08 – 2·19)	0·68	(0·18 – 1·20)
Female	9	52·2	10·3	54·2	10·7	71·5	14·1	34·9	6·9	1·30	(1·10 – 1·57)	**0·13**†	(-0·31 – 0·51)

Table 8: MAE and bias divided by patient monitor respiratory rate. The error is significantly higher at the <25 level. 95% confidence intervals are shown. Significant results are given in bold, where *, **, and *** indicate that the method performs worse (relative to all non-intervention data) on this subset (further from zero) at the p = 0.05, 0.005 and 0.0005 levels respectively, and †, ††, and ††† indicate that the method performs better on this subset (closer to zero) at the same levels.

Monitor RR	Fraction (%)	MAE	Bias
< 25	0·2	**7·57***** (5·53 – 12·53)	**7·44***** (5·41 – 12·33)
25 - 35	3·8	**1·98*** (1·42 – 4·52)	**1·71***** (1·14 – 4·07)
35 - 45	13·4	1·40 (1·12 – 2·01)	**0·91***** (0·64 – 1·38)
45 - 55	22·3	1·24 (0·97 – 1·87)	0·57 (0·34 – 1·08)
55 - 65	22·2	1·39 (1·04 – 1·95)	0·32 (-0·08 – 0·80)
65 - 75	19·9	1·48 (1·04 – 2·18)	**0·03**† (-0·60 – 0·48)
> 75	18·2	1·46 (1·07 – 1·96)	-0·41 (-0·95 – -0·06)
All	100·0	1·51 (1·18 – 1·87)	0·35 (0·02 – 0·65)

Table 9: Valid measurement time divided by activity/covering and patient monitor respiratory rate. All values are percentages. *, **, *** indicate that the value is significantly lower than that for the same subset, all RR at p=0·05, 0·005 and p=0·0005 levels; †, †† and ††† indicate significantly higher at the same levels.

Subset	RR	Monitor		Camera		Both		Either		Agreement	
All	< 25	25·8***	19·9 - 33·6	25·8***	18·5 - 35·4	6·5***	4·2 - 9·4	45·2***	38·8 - 52·9	61·3***	55·8 - 66·0
	25 - 35	33·2***	23·2 - 43·6	29·9***	21·6 - 38·2	15·0***	8·0 - 21·6	48·1***	38·4 - 56·2	66·9	63·3 - 71·4
	35 - 45	40·9	31·0 - 50·4	39·3*	30·1 - 47·6	25·0	16·5 - 33·2	55·1**	46·3 - 62·7	69·9	66·3 - 74·1
	45 - 55	48·1	39·4 - 56·9	47·7	37·4 - 57·5	32·9	22·8 - 43·7	62·9	55·9 - 69·8	69·9	65·7 - 74·8
	55 - 65	52·0†	44·4 - 59·1	53·8†	43·6 - 63·2	36·8†	27·6 - 46·1	68·9†	62·9 - 74·7	67·9	63·7 - 72·4
	65 - 75	54·8††	45·2 - 64·1	58·8††	48·2 - 68·4	40·4††	30·2 - 50·6	73·2†††	65·9 - 79·9	67·2	63·1 - 71·7
	> 75	48·8	35·6 - 63·2	56·3†	45·9 - 67·2	34·7	23·3 - 47·6	70·4††	61·9 - 79·3	64·4*	58·0 - 69·8
Intervention	< 25	29·4	20·9 - 42·2	29·6	13·5 - 39·3	7·2***	4·2 - 9·9	51·8††	44·1 - 57·3	55·3***	51·0 - 61·2
	25 - 35	25·0***	16·0 - 33·1	22·0*	11·1 - 31·5	9·9***	3·6 - 16·2	37·1**	26·9 - 44·9	72·9	68·6 - 77·2
	35 - 45	28·1*	17·8 - 36·9	23·5*	14·7 - 31·8	14·3*	5·9 - 22·4	37·4*	27·9 - 44·9	76·9††	72·5 - 80·8
	45 - 55	27·1*	20·7 - 34·1	23·6*	17·6 - 28·6	13·5*	9·0 - 17·8	37·1*	30·5 - 43·4	76·4†	72·3 - 79·8
	55 - 65	31·9	26·1 - 38·7	29·9	22·3 - 37·2	18·4	13·1 - 23·9	43·5	36·5 - 50·7	74·9	71·4 - 78·1
	65 - 75	43·4†††	34·7 - 52·5	39·4†††	28·7 - 49·8	27·6†††	18·7 - 36·5	55·1†††	45·7 - 64·5	72·5	70·4 - 74·7
	> 75	47·4†††	37·2 - 57·5	40·8†††	28·8 - 52·7	29·4†††	19·1 - 39·4	58·8†††	48·4 - 69·0	70·5*	67·0 - 73·4
Not Intervention	< 25	25·5***	19·8 - 33·4	25·6***	18·8 - 35·0	6·5***	4·3 - 9·7	44·6***	38·4 - 52·7	61·9**	56·1 - 66·4
	25 - 35	34·3***	24·2 - 45·0	31·0***	22·6 - 39·5	15·7***	8·6 - 22·7	49·5***	39·8 - 58·1	66·2	62·4 - 70·8
	35 - 45	42·8	32·7 - 52·8	41·7*	32·1 - 50·6	26·7	17·7 - 35·5	57·8**	48·8 - 65·7	68·9	65·1 - 73·3
	45 - 55	51·0	41·8 - 60·3	51·1	40·0 - 61·5	35·5	24·7 - 47·2	66·5	59·4 - 73·5	69·0	64·2 - 74·4
	55 - 65	54·5†	46·6 - 61·9	56·8†	45·9 - 66·8	39·2†	29·3 - 49·0	72·2††	66·0 - 78·0	67·0	62·4 - 72·0
	65 - 75	56·2†	46·3 - 65·6	61·2††	50·1 - 71·2	42·0†	31·4 - 52·6	75·5†††	67·9 - 82·1	66·5	62·0 - 71·6
	> 75	48·9	35·0 - 64·5	58·1†	47·1 - 69·8	35·4	23·3 - 49·6	71·7†	62·6 - 81·3	63·6	56·7 - 69·6
Uncovered	< 25	24·7***	18·0 - 40·3	30·5***	22·3 - 43·1	8·0***	4·7 - 13·7	47·2***	38·9 - 59·1	60·8***	52·7 - 66·6
	25 - 35	34·7**	23·1 - 49·3	38·6***	28·3 - 49·0	20·7***	10·4 - 31·1	52·7***	41·4 - 63·7	67·9	65·8 - 71·2
	35 - 45	43·2	31·1 - 55·6	50·1*	40·0 - 59·5	32·1	20·3 - 43·8	61·2*	51·4 - 69·8	70·9	66·9 - 76·2
	45 - 55	51·7	37·3 - 65·4	60·2	50·2 - 69·2	42·2	27·7 - 56·5	69·8	60·8 - 77·8	72·4	65·7 - 79·2
	55 - 65	53·4	41·7 - 64·1	65·5††	58·6 - 71·3	44·5†	33·3 - 54·9	74·4†	68·0 - 80·0	70·0	64·5 - 76·0
	65 - 75	54·7†	44·0 - 66·3	68·8†††	61·9 - 74·9	45·9†	36·0 - 57·0	77·5†††	71·5 - 83·4	68·5	63·1 - 74·6
	> 75	48·6	35·5 - 64·0	65·0††	54·1 - 74·9	40·9	27·9 - 56·0	72·6†	62·6 - 82·4	68·3	62·2 - 74·5
Covered	< 25	26·5***	22·6 - 30·8	19·3***	15·5 - 27·1	4·5***	3·2 - 7·1	41·3***	37·1 - 48·0	63·2	58·1 - 66·8
	25 - 35	33·7***	23·0 - 41·4	20·1***	16·1 - 24·2	8·7***	6·0 - 11·6	45·0***	34·9 - 52·0	63·7	58·5 - 72·0
	35 - 45	42·3	31·6 - 51·6	29·2**	21·7 - 37·4	18·7*	12·0 - 25·6	52·8***	42·9 - 61·6	65·8	61·4 - 71·4
	45 - 55	50·1	44·2 - 56·5	40·0	29·8 - 52·1	27·5	19·5 - 37·4	62·6	56·6 - 69·8	64·9	61·3 - 69·0
	55 - 65	55·6†	48·8 - 62·9	48·4	36·8 - 61·8	34·0†	24·6 - 45·9	70·0†	64·1 - 77·0	64·1	59·4 - 69·9
	65 - 75	57·5††	47·7 - 67·8	54·7†	42·7 - 67·4	38·5††	27·9 - 50·9	73·7†††	66·1 - 81·9	64·8	60·2 - 70·5
	> 75	49·2	33·7 - 67·3	52·3†	40·2 - 64·9	30·6	19·2 - 45·8	70·9†	62·9 - 80·6	59·7*	52·6 - 67·4
Inactive	< 25	26·1***	19·9 - 32·8	29·8***	19·6 - 41·4	8·3***	5·1 - 11·3	47·6***	38·9 - 55·8	60·7*	54·2 - 67·2
	25 - 35	37·0**	26·6 - 45·9	37·4**	25·6 - 47·7	19·9**	10·4 - 27·0	54·4***	43·5 - 62·1	65·5	62·5 - 68·9
	35 - 45	48·1	37·1 - 57·7	50·5	35·8 - 62·1	34·0	20·6 - 45·1	64·7*	54·1 - 72·8	69·3	64·8 - 74·0
	45 - 55	57·5	46·4 - 68·6	58·5	42·7 - 71·5	42·8	27·3 - 57·7	73·2	64·2 - 81·3	69·7	62·3 - 76·8
	55 - 65	59·8†	50·9 - 67·7	60·8	45·8 - 74·4	44·0	30·7 - 56·9	76·5†	69·2 - 83·5	67·5	61·0 - 74·0
	65 - 75	60·5†	48·1 - 70·9	63·1	48·1 - 77·1	45·1†	30·9 - 59·4	78·5††	70·2 - 86·2	66·6	59·8 - 74·1
	> 75	50·3	32·8 - 69·3	58·9	44·0 - 74·3	34·7	20·4 - 53·0	74·5	66·8 - 84·7	60·2*	51·2 - 69·7
Active	< 25	24·9***	19·2 - 36·3	20·9***	15·3 - 26·1	4·5***	3·5 - 6·7	41·3***	36·3 - 50·2	63·2**	56·1 - 68·0
	25 - 35	31·6***	21·0 - 45·1	24·7***	19·2 - 29·5	11·6***	7·0 - 17·5	44·7***	34·7 - 54·8	66·9	61·2 - 73·8
	35 - 45	37·5	26·3 - 51·3	32·8***	27·0 - 37·5	19·4**	13·6 - 26·0	50·9**	41·1 - 61·3	68·5	63·6 - 74·1
	45 - 55	43·7	36·2 - 52·0	42·9	35·4 - 50·2	27·4	21·0 - 34·6	59·1	53·3 - 65·4	68·3	64·9 - 72·2
	55 - 65	47·9	40·6 - 55·3	51·8†	43·5 - 59·6	33·1†	26·4 - 40·4	66·6†	61·2 - 72·1	66·4	63·3 - 70·3
	65 - 75	50·7†	41·7 - 60·4	58·9†††	49·8 - 66·6	38·0†††	29·9 - 46·2	71·6†††	63·6 - 78·5	66·4	63·9 - 69·4
	> 75	47·2	32·7 - 62·7	57·2†††	46·6 - 68·2	36·2††	23·5 - 49·1	68·3††	56·5 - 79·7	67·9	64·8 - 70·9
Uncovered, Inactive	< 25	27·2***	18·8 - 41·8	36·0***	22·6 - 48·3	10·8***	6·3 - 15·8	52·4***	39·7 - 62·4	58·4***	50·6 - 67·8
	25 - 35	39·9*	27·3 - 51·6	47·6***	34·1 - 56·5	27·4**	14·4 - 36·2	60·2***	47·7 - 68·0	67·2	64·0 - 70·9
	35 - 45	51·7	36·0 - 63·5	62·3	47·9 - 70·3	42·9	26·1 - 54·6	71·0	58·2 - 78·1	71·9	66·4 - 78·1
	45 - 55	61·2	43·2 - 75·4	70·6†	58·0 - 79·0	53·3†	34·4 - 68·0	78·5†	67·4 - 86·2	74·8†	65·6 - 82·2
	55 - 65	61·0	47·2 - 72·2	72·4††	64·2 - 78·6	52·9†	39·1 - 64·2	80·5†	73·1 - 86·2	72·4	65·3 - 78·6
	65 - 75	60·1	45·4 - 73·2	73·7††	66·3 - 80·3	52·1	38·1 - 65·0	81·7††	74·9 - 87·7	70·4	62·2 - 77·9
	> 75	47·0	31·8 - 71·1	67·8	56·9 - 81·1	40·6	26·1 - 63·6	74·2	63·9 - 87·6	66·4	57·5 - 76·5
Uncovered, Active	< 25	21·9***	16·5 - 40·7	24·3***	15·6 - 32·0	4·9***	3·6 - 9·3	41·3***	36·0 - 53·1	63·5***	54·6 - 69·6
	25 - 35	29·4***	19·4 - 47·4	29·4***	23·4 - 37·0	13·8***	7·7 - 23·7	45·1***	35·4 - 58·4	68·7	63·9 - 74·5
	35 - 45	34·5	24·6 - 49·5	37·6**	33·4 - 42·8	21·1*	14·9 - 30·1	51·1**	43·9 - 61·6	69·9	66·3 - 74·8
	45 - 55	41·5	31·2 - 52·2	49·2	42·4 - 55·0	30·3	21·3 - 39·3	60·4	53·7 - 66·9	69·9	65·4 - 75·2
	55 - 65	45·1	35·5 - 54·8	58·0††	50·8 - 63·8	35·3†	26·8 - 43·5	67·8†	61·4 - 73·9	67·5	63·3 - 73·1
	65 - 75	48·9†	40·8 - 58·7	63·6†††	54·8 - 70·2	39·4††	32·1 - 48·0	73·0†††	66·3 - 79·6	66·4	63·2 - 71·3
	> 75	50·3†	39·9 - 59·9	61·8†††	48·5 - 71·4	41·2†††	29·6 - 51·1	70·9††	59·3 - 79·5	70·3	67·5 - 73·7
Covered, Inactive	< 25	24·6***	20·5 - 28·6	21·9***	15·9 - 31·8	5·1***	3·4 - 7·6	41·4***	36·7 - 48·7	63·7	57·7 - 67·7
	25 - 35	32·7***	24·4 - 39·0	22·3***	17·2 - 28·4	9·0***	6·2 - 12·0	45·9***	38·6 - 52·1	63·1	58·4 - 69·0
	35 - 45	42·8*	35·0 - 49·4	32·8*	22·6 - 44·0	20·4*	12·5 - 29·1	55·2***	47·8 - 62·7	65·2	61·5 - 69·3
	45 - 55	53·2	47·3 - 58·9	44·4	29·6 - 60·2	30·6	19·5 - 43·1	66·9	60·1 - 74·8	63·7	58·5 - 69·3
	55 - 65	58·8††	51·4 - 66·2	50·9	35·4 - 68·9	36·5	24·4 - 51·5	73·2†	66·5 - 81·3	63·3	57·2 - 70·9
	65 - 75	60·7†††	49·2 - 72·0	55·1	39·9 - 72·5	39·8†	27·0 - 55·9	76·1††	68·0 - 85·3	63·7	57·8 - 71·8
	> 75	52·9	32·3 - 72·2	52·0	35·0 - 69·9	30·1	16·3 - 51·1	74·7††	68·0 - 84·3	55·4**	47·5 - 67·5
Covered, Active	< 25	28·8***	23·7 - 39·0	16·5***	13·7 - 20·7	4·1***	3·0 - 6·6	41·2***	35·3 - 52·2	62·8	53·1 - 68·5
	25 - 35	34·7**	21·4 - 44·8	18·0***	13·4 - 21·1	8·5***	5·3 - 12·0	44·2***	30·3 - 53·0	64·3	58·0 - 75·6
	35 - 45	41·7	26·1 - 54·9	25·8***	19·5 - 31·6	17·0**	10·5 - 22·9	50·5**	36·1 - 62·4	66·4	59·6 - 75·3
	45 - 55	46·5	40·1 - 54·7	34·9	29·2 - 43·9	23·8	18·9 - 31·5	57·6	51·8 - 65·4	66·3	62·9 - 70·0
	55 - 65	51·0	44·3 - 58·8	44·9†	37·8 - 55·0	30·6†	24·4 - 39·7	65·4†	60·2 - 72·1	65·2	62·2 - 69·1
	65 - 75	52·7†	41·2 - 64·6	53·9†††	45·3 - 63·6	36·6††	27·2 - 47·0	70·1††	61·1 - 79·3	66·5	64·0 - 69·3
	> 75	44·3	27·5 - 65·8	52·8†††	44·6 - 64·5	31·3†	19·2 - 47·4	65·7†	54·1 - 80·4	65·6	61·7 - 69·1

Table 10: MAE and bias divided by day/night shift. We define day shift as 8am-8pm and night otherwise. 95% confidence intervals are shown. Significant results are given in bold, where *, **, and *** indicate that the method performs worse (relative to all non-intervention data) on this subset (further from zero) at the p = 0.05, 0.005 and 0.0005 levels respectively, and †, ††, and ††† indicate that the method performs better on this subset (closer to zero) at the same levels.

	Breath Matching				Rate Comparison			
	Day		Night		Day		Night	
Subset	MAE	Bias	MAE	Bias	MAE	Bias	MAE	Bias
All	1·54<br>(1·24 – 1·92)	0·40<br>(0·04 – 0·78)	1·50<br>(1·18 – 1·91)	0·52<br>(0·10 – 0·95)	1·53<br>(1·16 – 1·87)	0·41<br>(0·13 – 0·72)	1·63<br>(1·22 – 2·12)	0·35<br>(-0·14 – 0·73)
Intervention	2·28<br>(1·70 – 3·07)	0·34<br>(-0·23 – 0·85)	2·66<br>(1·84 – 3·47)	1·67<br>(0·55 – 2·84)	2·57<br>(1·58 – 3·62)	0·35<br>(-0·38 – 0·96)	2·85<br>(1·69 – 3·60)	1·88<br>(0·55 – 2·79)
Not-Intervention	1·46<br>(1·16 – 1·82)	0·40<br>(0·03 – 0·80)	1·43<br>(1·14 – 1·79)	0·44<br>(0·06 – 0·82)	1·43<br>(1·12 – 1·74)	0·42<br>(0·14 – 0·73)	1·58<br>(1·19 – 2·05)	0·29<br>(-0·19 – 0·64)
Uncovered	1·51<br>(1·07 – 2·07)	0·57<br>(0·06 – 1·08)	1·45<br>(1·09 – 1·91)	0·59<br>(0·18 – 1·02)	1·45<br>(1·04 – 1·95)	0·61<br>(0·26 – 1·00)	1·42<br>(1·07 – 1·88)	0·57<br>(0·29 – 0·92)
Covered	1·37<br>(1·13 – 1·64)	0·17<br>(-0·18 – 0·52)	1·41<br>(1·17 – 1·70)	0·24<br>(-0·15 – 0·59)	**1·39**†<br>(1·12 – 1·61)	0·14<br>(-0·14 – 0·45)	**1·84*****<br>(1·34 – 2·31)	-0·16<br>(-0·81 – 0·35)
Inactive	1·22<br>(0·95 – 1·57)	0·21<br>(-0·20 – 0·57)	1·19<br>(1·00 – 1·45)	0·28<br>(-0·09 – 0·61)	1·19<br>(0·95 – 1·47)	0·29<br>(0·05 – 0·53)	1·35<br>(1·01 – 1·92)	0·10<br>(-0·50 – 0·47)
Active	1·76<br>(1·36 – 2·23)	0·64<br>(0·17 – 1·16)	1·78<br>(1·36 – 2·23)	0·68<br>(0·11 – 1·23)	1·80<br>(1·34 – 2·26)	0·62<br>(0·21 – 1·10)	2·01<br>(1·48 – 2·46)	0·63<br>(0·11 – 1·10)
Uncovered Inactive	1·26<br>(0·90 – 1·78)	0·39<br>(-0·13 – 0·83)	1·15<br>(0·92 – 1·48)	0·21<br>(-0·18 – 0·53)	1·16<br>(0·92 – 1·56)	0·44<br>(0·22 – 0·72)	1·06<br>(0·89 – 1·29)	0·31<br>(0·11 – 0·49)
Uncovered Active	1·90<br>(1·28 – 2·59)	0·81<br>(0·15 – 1·52)	1·83<br>(1·30 – 2·37)	1·08<br>(0·55 – 1·62)	1·98<br>(1·27 – 2·73)	0·92<br>(0·28 – 1·61)	2·03<br>(1·42 – 2·67)	1·03<br>(0·49 – 1·61)
Covered Inactive	1·15<br>(0·93 – 1·36)	-0·09<br>(-0·47 – 0·29)	1·25<br>(1·08 – 1·48)	0·36<br>(-0·01 – 0·72)	1·22<br>(0·94 – 1·45)	0·02<br>(-0·34 – 0·38)	**1·77*****<br>(1·17 – 2·53)	-0·20<br>(-1·08 – 0·50)
Covered Active	1·59<br>(1·26 – 1·99)	0·43<br>(-0·03 – 0·89)	1·70<br>(1·33 – 2·13)	**0·04**†<br>(-0·51 – 0·58)	1·59<br>(1·25 – 1·94)	0·28<br>(-0·03 – 0·63)	**1·96***<br>(1·50 – 2·25)	-0·07<br>(-0·54 – 0·32)

Table 11: Main results with varied window size for evaluation. The window size l is given in seconds. Increasing the window size reduces the errors as extra and missed breaths within the same window cancel out. 95% confidence intervals are shown; significant results are given in bold, where *, **, and *** indicate that the method performs worse than the 12s window at the p = 0.05, 0.005 and 0.0005 levels respectively, and †, ††, and ††† indicate that the method performs better at the same levels.

		Breath Matching		Rate Comparison	
Subset	l	MAE	Bias	MAE	Bias
All	6	1·80 (1·44 – 2·25)	0·56 (0·16 – 0·99)	**2·41***** (1·97 – 2·96)	0·50 (0·12 – 0·93)
	8	1·67 (1·33 – 2·08)	0·51 (0·13 – 0·90)	**1·98*** (1·59 – 2·46)	0·45 (0·12 – 0·82)
	10	1·58 (1·26 – 1·98)	0·48 (0·12 – 0·86)	1·72 (1·37 – 2·17)	0·41 (0·09 – 0·74)
	12	1·52 (1·21 – 1·90)	0·46 (0·11 – 0·82)	1·54 (1·21 – 1·96)	0·37 (0·05 – 0·68)
	14	1·48 (1·18 – 1·85)	0·45 (0·11 – 0·80)	1·42 (1·11 – 1·83)	0·33 (-0·00 – 0·63)
	18	1·41 (1·13 – 1·76)	0·43 (0·10 – 0·76)	1·29 (0·98 – 1·71)	0·23 (-0·15 – 0·54)
Intervention	6	3·03 (2·29 – 3·91)	1·09 (0·38 – 1·81)	**3·86*** (2·83 – 5·06)	0·98 (0·23 – 1·71)
	8	2·74 (2·05 – 3·58)	0·97 (0·31 – 1·63)	3·20 (2·22 – 4·38)	0·90 (0·26 – 1·55)
	10	2·55 (1·88 – 3·37)	0·88 (0·26 – 1·51)	2·77 (1·87 – 3·89)	0·82 (0·26 – 1·40)
	12	2·41 (1·76 – 3·22)	0·83 (0·23 – 1·43)	2·47 (1·62 – 3·61)	0·76 (0·29 – 1·28)
	14	2·31 (1·67 – 3·10)	0·79 (0·21 – 1·38)	2·24 (1·42 – 3·41)	0·74 (0·29 – 1·22)
	18	2·16 (1·54 – 2·93)	0·73 (0·18 – 1·28)	1·96 (1·19 – 3·13)	0·64 (0·16 – 1·10)
Not Intervention	6	1·69 (1·36 – 2·11)	0·51 (0·11 – 0·93)	**2·31***** (1·90 – 2·82)	0·46 (0·08 – 0·89)
	8	1·57 (1·26 – 1·96)	0·47 (0·09 – 0·86)	**1·89*** (1·54 – 2·34)	0·42 (0·08 – 0·78)
	10	1·49 (1·20 – 1·86)	0·44 (0·08 – 0·81)	1·65 (1·33 – 2·07)	0·38 (0·05 – 0·71)
	12	1·44 (1·16 – 1·80)	0·42 (0·08 – 0·78)	1·48 (1·18 – 1·87)	0·34 (0·02 – 0·65)
	14	1·40 (1·13 – 1·75)	0·41 (0·07 – 0·76)	1·37 (1·08 – 1·75)	0·30 (-0·04 – 0·60)
	18	1·34 (1·08 – 1·67)	0·40 (0·07 – 0·73)	1·25 (0·95 – 1·65)	0·21 (-0·19 – 0·52)
Uncovered	6	1·72 (1·26 – 2·31)	0·70 (0·20 – 1·23)	**2·27**** (1·73 – 2·99)	0·72 (0·29 – 1·28)
	8	1·60 (1·17 – 2·16)	0·64 (0·17 – 1·14)	**1·85*** (1·39 – 2·48)	0·65 (0·29 – 1·13)
	10	1·53 (1·11 – 2·06)	0·60 (0·15 – 1·09)	1·60 (1·20 – 2·16)	0·61 (0·29 – 1·03)
	12	1·48 (1·08 – 1·99)	0·58 (0·13 – 1·05)	1·41 (1·06 – 1·92)	0·58 (0·30 – 0·95)
	14	1·44 (1·05 – 1·94)	0·56 (0·13 – 1·02)	1·28 (0·96 – 1·73)	0·55 (0·29 – 0·88)
	18	1·38 (1·01 – 1·86)	0·54 (0·12 – 0·98)	1·11 (0·85 – 1·50)	0·49 (0·25 – 0·78)
Covered	6	**1·66*** (1·38 – 1·98)	0·26 (-0·08 – 0·59)	**2·36***** (2·02 – 2·77)	0·09 (-0·27 – 0·44)
	8	1·53 (1·28 – 1·82)	0·23 (-0·08 – 0·54)	**1·96*** (1·65 – 2·31)	0·08 (-0·27 – 0·40)
	10	1·45 (1·22 – 1·72)	0·22 (-0·08 – 0·51)	1·73 (1·44 – 2·08)	0·03 (-0·35 – 0·36)
	12	1·39 (1·17 – 1·65)	0·21 (-0·08 – 0·49)	1·59 (1·28 – 1·95)	-0·01 (-0·43 – 0·34)
	14	1·35 (1·14 – 1·59)	0·21 (-0·08 – 0·48)	1·50 (1·17 – 1·92)	-0·07 (-0·55 – 0·31)
	18	1·29 (1·09 – 1·51)	0·20 (-0·07 – 0·47)	1·45 (1·04 – 1·99)	-0·20 (-0·81 – 0·27)
Inactive	6	1·38 (1·13 – 1·71)	0·30 (-0·08 – 0·65)	**1·91**** (1·62 – 2·27)	0·28 (-0·04 – 0·58)
	8	1·30 (1·07 – 1·60)	0·27 (-0·09 – 0·60)	**1·58*** (1·32 – 1·91)	0·25 (-0·06 – 0·53)
	10	1·24 (1·02 – 1·54)	0·26 (-0·10 – 0·58)	1·39 (1·14 – 1·74)	0·22 (-0·11 – 0·49)
	12	1·20 (0·99 – 1·49)	0·25 (-0·10 – 0·56)	1·27 (1·01 – 1·64)	0·19 (-0·17 – 0·46)
	14	1·17 (0·97 – 1·45)	0·24 (-0·10 – 0·55)	1·19 (0·92 – 1·59)	0·15 (-0·26 – 0·43)
	18	1·13 (0·94 – 1·40)	0·24 (-0·09 – 0·54)	1·12 (0·82 – 1·62)	0·07 (-0·44 – 0·40)
Active	6	2·12 (1·65 – 2·62)	0·80 (0·29 – 1·32)	**2·89***** (2·32 – 3·53)	0·74 (0·19 – 1·35)
	8	1·95 (1·52 – 2·41)	0·73 (0·26 – 1·22)	**2·38*** (1·87 – 2·95)	0·68 (0·21 – 1·20)
	10	1·84 (1·43 – 2·29)	0·69 (0·24 – 1·16)	2·07 (1·60 – 2·60)	0·63 (0·20 – 1·10)
	12	1·77 (1·37 – 2·20)	0·66 (0·23 – 1·11)	1·84 (1·41 – 2·32)	0·60 (0·20 – 1·02)
	14	1·71 (1·33 – 2·13)	0·64 (0·22 – 1·08)	1·68 (1·29 – 2·12)	0·57 (0·19 – 0·95)
	18	1·63 (1·26 – 2·03)	0·61 (0·21 – 1·03)	1·47 (1·14 – 1·86)	0·48 (0·11 – 0·85)
Uncovered, Inactive	6	1·37 (1·03 – 1·86)	0·37 (-0·11 – 0·78)	**1·78***** (1·47 – 2·26)	0·43 (0·13 – 0·75)
	8	1·29 (0·98 – 1·75)	0·33 (-0·12 – 0·72)	**1·44*** (1·19 – 1·84)	0·41 (0·17 – 0·68)
	10	1·24 (0·94 – 1·68)	0·31 (-0·14 – 0·69)	1·24 (1·03 – 1·59)	0·39 (0·19 – 0·63)
	12	1·21 (0·91 – 1·64)	0·30 (-0·14 – 0·67)	1·11 (0·92 – 1·42)	0·38 (0·20 – 0·59)
	14	1·18 (0·90 – 1·60)	0·30 (-0·14 – 0·66)	1·01 (0·85 – 1·29)	0·36 (0·20 – 0·56)
	18	1·14 (0·87 – 1·55)	0·29 (-0·13 – 0·64)	**0·90**† (0·76 – 1·14)	0·32 (0·16 – 0·49)
Uncovered, Active	6	2·20 (1·54 – 2·88)	1·14 (0·50 – 1·78)	**3·01**** (2·21 – 3·85)	1·15 (0·48 – 1·90)
	8	2·03 (1·42 – 2·67)	1·04 (0·45 – 1·65)	2·50 (1·78 – 3·29)	1·04 (0·44 – 1·70)
	10	1·92 (1·33 – 2·55)	0·99 (0·41 – 1·56)	2·19 (1·51 – 2·92)	0·98 (0·42 – 1·57)
	12	1·85 (1·28 – 2·45)	0·94 (0·39 – 1·50)	1·96 (1·35 – 2·64)	0·94 (0·43 – 1·49)
	14	1·80 (1·24 – 2·39)	0·91 (0·37 – 1·45)	1·78 (1·22 – 2·40)	0·90 (0·42 – 1·40)
	18	1·71 (1·18 – 2·28)	0·86 (0·35 – 1·38)	1·55 (1·06 – 2·11)	0·82 (0·37 – 1·32)
Covered, Inactive	6	**1·41*** (1·22 – 1·62)	0·21 (-0·16 – 0·58)	**2·09*** (1·83 – 2·39)	0·06 (-0·33 – 0·45)
	8	1·31 (1·14 – 1·51)	0·19 (-0·16 – 0·53)	1·78 (1·49 – 2·11)	0·03 (-0·39 – 0·43)
	10	1·25 (1·09 – 1·44)	0·18 (-0·16 – 0·51)	1·62 (1·29 – 2·01)	-0·03 (-0·51 – 0·40)
	12	1·21 (1·05 – 1·40)	0·17 (-0·16 – 0·50)	1·51 (1·14 – 1·99)	-0·10 (-0·65 – 0·37)
	14	1·18 (1·02 – 1·36)	0·17 (-0·15 – 0·49)	1·46 (1·02 – 2·02)	-0·18 (-0·82 – 0·34)
	18	1·13 (0·98 – 1·30)	0·17 (-0·14 – 0·48)	1·47 (0·91 – 2·23)	-0·32 (-1·16 – 0·33)
Covered, Active	6	**1·99*** (1·58 – 2·43)	0·33 (-0·02 – 0·67)	**2·73***** (2·21 – 3·35)	0·15 (-0·23 – 0·52)
	8	1·82 (1·45 – 2·22)	0·30 (-0·02 – 0·62)	**2·22*** (1·79 – 2·74)	0·16 (-0·17 – 0·48)
	10	1·71 (1·37 – 2·08)	0·28 (-0·02 – 0·58)	1·90 (1·53 – 2·36)	0·14 (-0·16 – 0·43)
	12	1·63 (1·32 – 1·98)	0·27 (-0·02 – 0·56)	1·68 (1·35 – 2·09)	0·14 (-0·16 – 0·40)
	14	1·58 (1·27 – 1·91)	0·26 (-0·02 – 0·55)	1·55 (1·24 – 1·94)	0·12 (-0·18 – 0·38)
	18	1·50 (1·22 – 1·80)	0·26 (-0·01 – 0·53)	**1·39**† (1·11 – 1·76)	0·04 (-0·30 – 0·32)

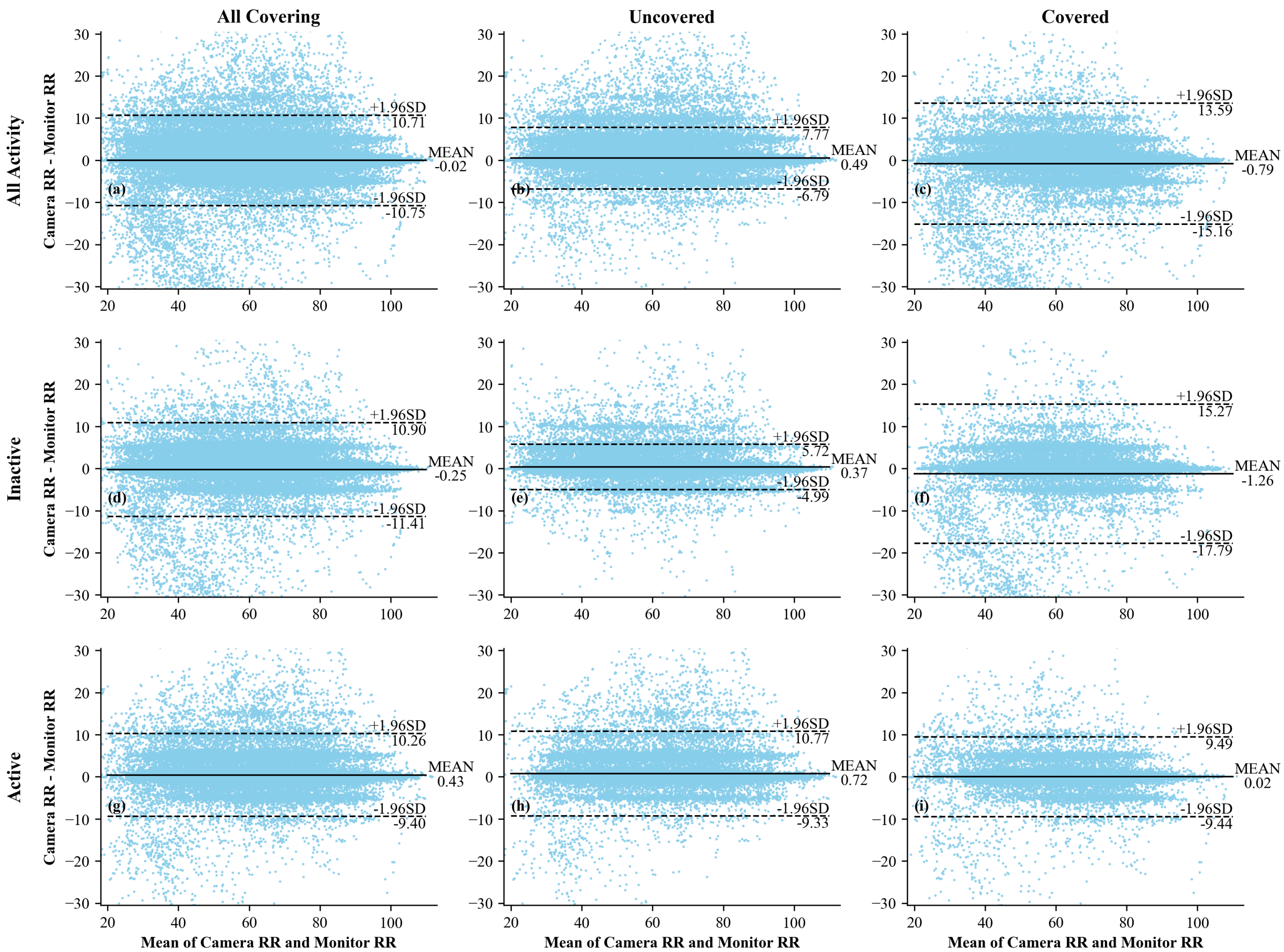

Figure 10: Bland-Altman plots of respiratory rate measurement for non-intervention data, divided into covering and activity. All measurements are given in bpm. Values are concentrated around zero and ±5 because the rate is calculated using a 12-second window; if either camera or monitor fails to detect a breath in this period, the error will be 5 bpm.

10 ECG Waveform Analysis

This section describes analysis of the patient monitor's ECG waveform quality, compared to the camera and patient monitor's respiratory waveform. The SQIs are designed to detect when the extracted signal is poor quality and fails to represent the patient's respiratory rate, which we expect is usually due to motion. We can establish a measure of the baby's activity using the torso position, which we find as part of the signal extraction process. The activity at a given time is the mean torso joint position, though to reduce noise we exclude the joint with the largest movement in each time-step. It is unlikely that only one joint is moved, so this rejects spurious joint positions from the pose estimation.

To compare the activity with the SQIs, we calculate the range of SQI values when the activity is high or low, as determined by a threshold based on the range of activity levels in each recording segment. For example, setting the threshold at 30% means we compare the SQIs for camera and patient monitor in the lowest 30% activity times and the highest 70% activity times.

We introduce the ECG analysis because there is an inherent link between the camera SQI and activity level – if the pose estimation performs poorly, we should expect high activity (due to noise) and poor camera signal quality (due to the wrong regions being selected). The ECG waveform is, in general, better quality than the respiratory waveform. We calculate an SQI for the ECG using the template matching method[11] implemented in the NeuroKit Python package,[12] which we compare to the activity.

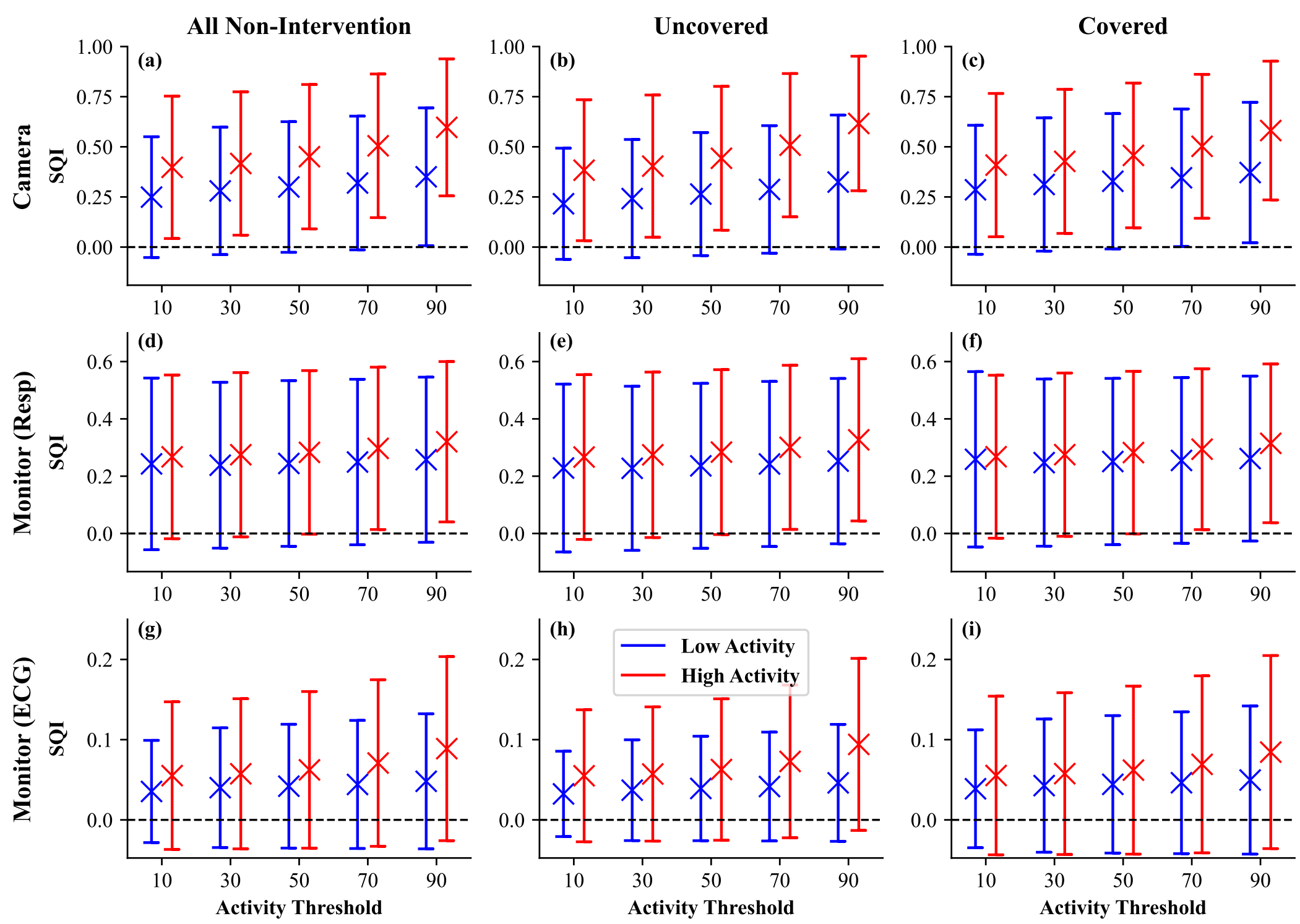

Figure 11: Comparison of SQI ranges and patient activity levels. The activity threshold X is set separately for each recording segment such that X% of the activity falls below the threshold. Higher SQI indicates a poor quality signal. Each line indicates mean (marked with a cross) and standard deviation (height). The camera shows increased SQI for higher activity thresholds (**a**-**c**); this is much less noticeable for the patient monitor's respiratory waveform (**d**-**f**). However, the ECG waveform shows some SQI increase with activity (**g**-**i**), though much more so when the baby is uncovered (**h**).

The relationship between SQI and activity is shown in Figure 11. The camera signal is typically worse when the baby is more active, though it is noted that activity is also measured using the camera. However, the ECG is typically worse during periods of high activity when the baby is covered, suggesting that these periods are often movement. This relationship is less notable when the baby is covered, suggesting that some 'activity' is caused by poor pose estimation with less visible patients. The relationship between monitor SQI and activity is much less pronounced, even when the baby is uncovered, suggesting that there are other sources of poor monitor signal quality. This leads us to conclude that:

- The decrease in camera and ECG signal quality with activity when the baby is uncovered indicates that activity causes poor uncovered signal quality.

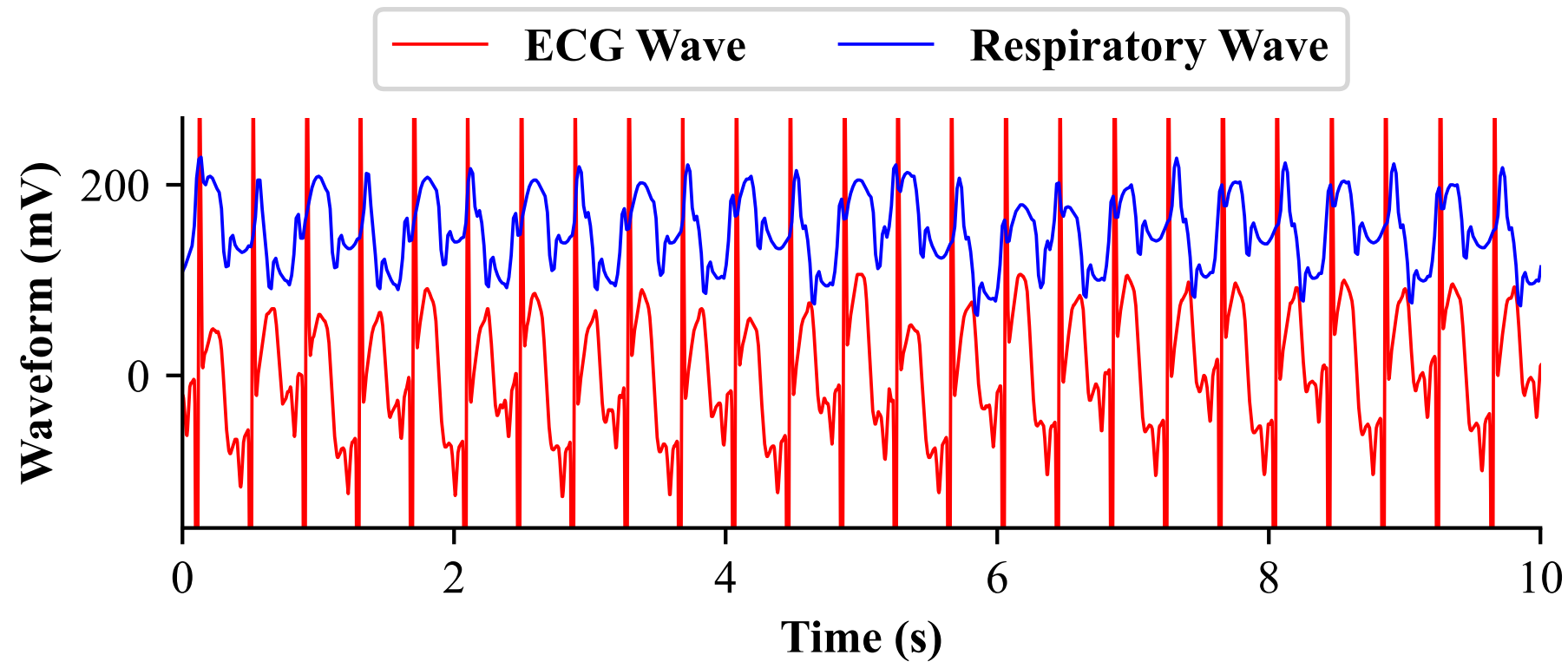

Figure 12: Example of the patient monitor respiratory wave including the patient's ECG. The respiratory wave is offset by 150mV and the Q/R waves are clipped to improve visual clarity. Both respiratory rate and heart rate were recorded as 149 breaths/beats per minute. The T wave from the ECG is visible in the respiratory wave, leading to an incorrect measurement.

- The decrease in camera signal quality with activity when covered, which is not consistent with the ECG, may be caused by poor pose estimation, which we know to be worse when the baby is covered.[13]
- The relationship between signal quality and activity is less notable for the patient monitor, indicating that other factors affect the patient monitor's signal quality. This matches with the low SQI agreement (around 65%) between monitor and camera, and our observation that the monitor may erroneously measure heart rate (illustrated in Figure 12. Some poor signal quality will also arise from signal processing errors.

11 Sample Size Calculation

Figures 13 and 14 show the relationship between the number of participants in the dataset and the 95% confidence intervals around the statistics of interest, mean absolute error and bias. These calculations are performed after collecting the data, as some of the relevant information - the size of each subset and the distribution of the errors within and between participants - is not known a priori. The calculations are repeated for each subset in the data; the uncovered + active subset has the widest confidence intervals, excluding interventions. The number of participants required to reduce the MAE upper bound to 2.5bpm and the bias upper bound to 0.5bpm above the mean is also shown; this is based on an extrapolation which assumes our data is representative of all patients. Excluding interventions, which account for a comparatively small volume of data, we might suggest that around 40 participants should be included to achieve the specified intervals for the uncovered + active subset.

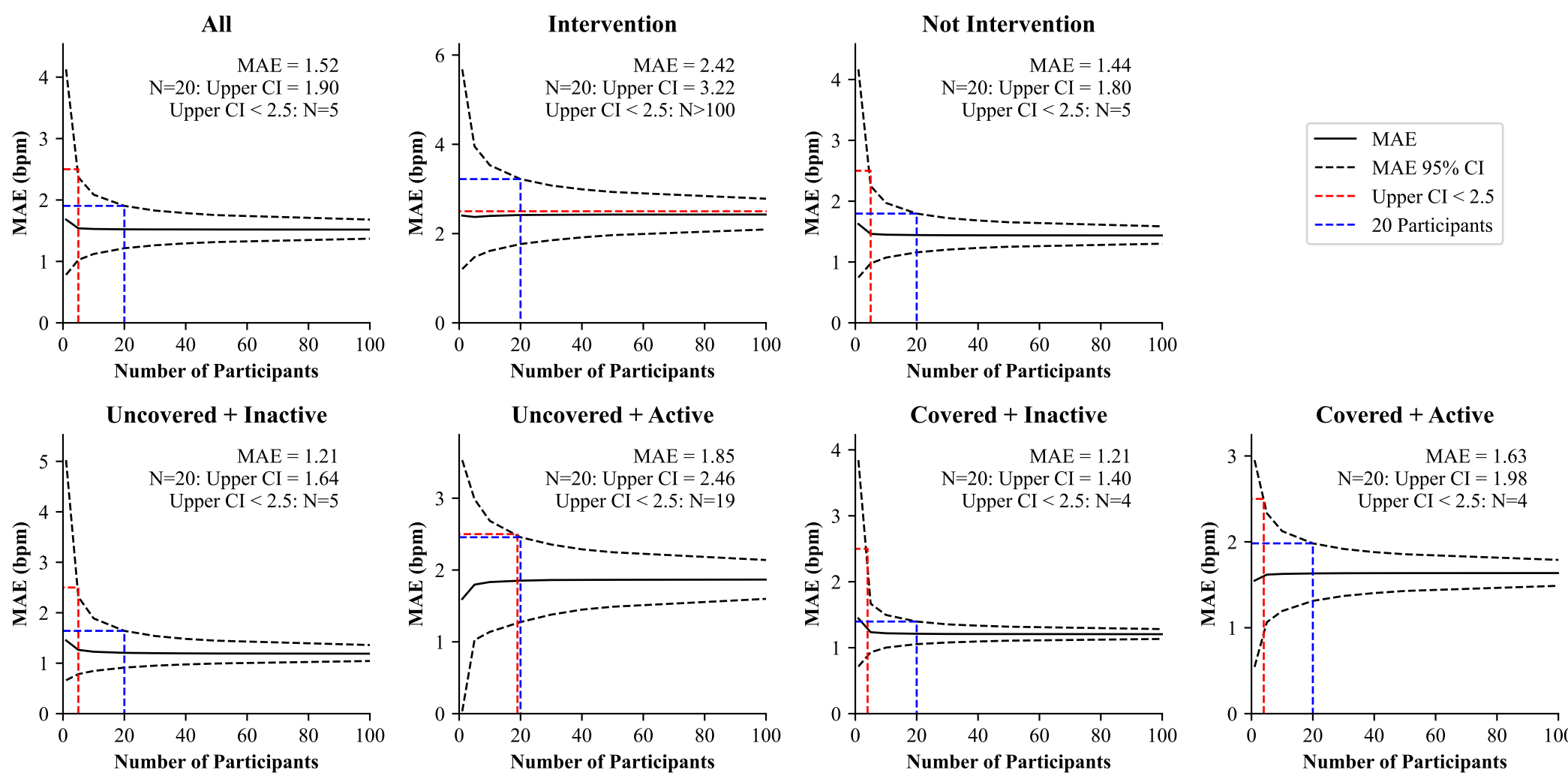

Figure 13: Confidence intervals for mean absolute error (MAE) as the number of participants is increased. $N > 20$ is an extrapolation. The confidence interval for the presented data is given in blue; the predicted number of samples for an upper CI < 2.5 is given in red.

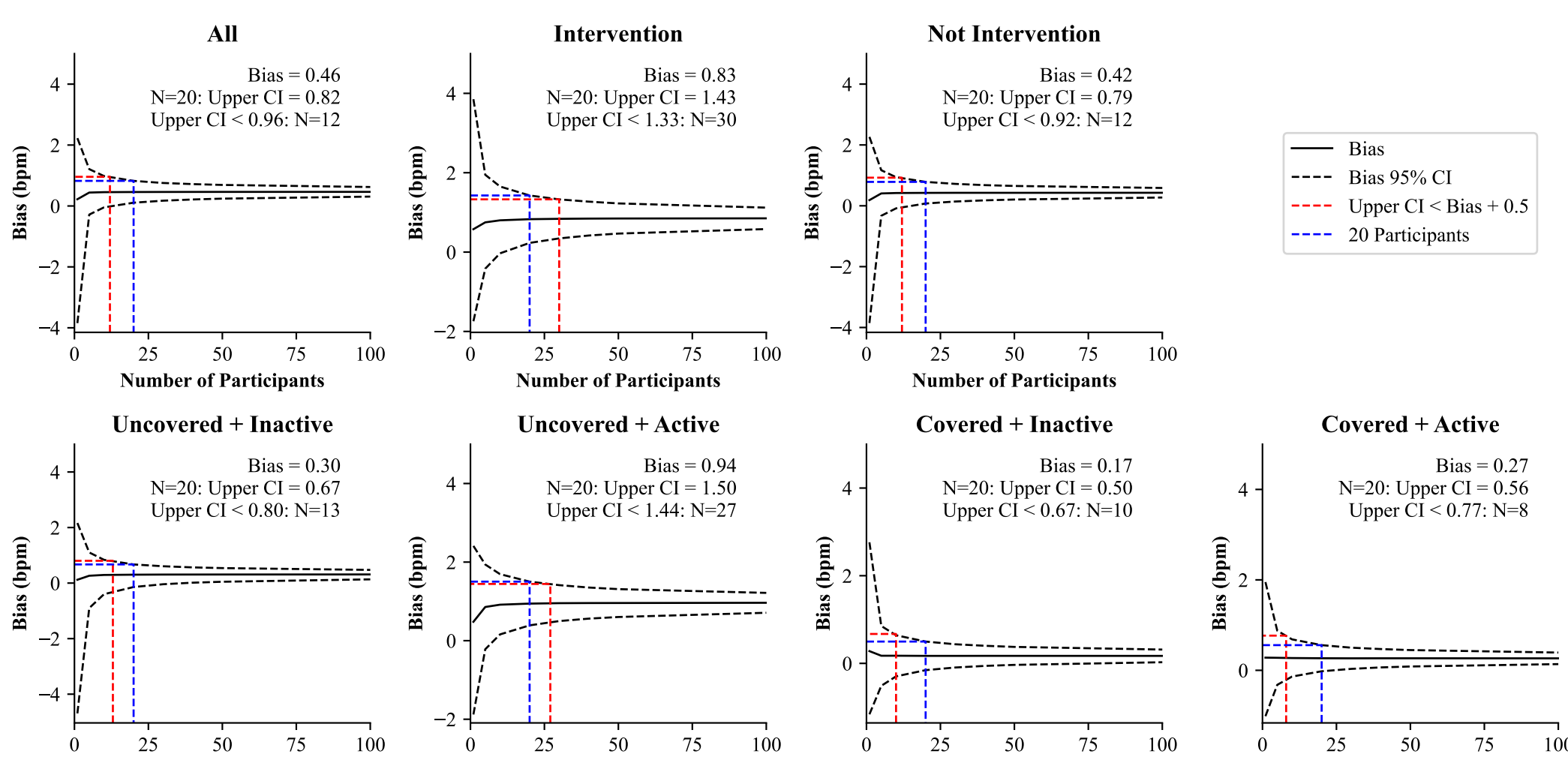

Figure 14: Confidence intervals for the bias as the number of participants is increased. $N > 20$ is an extrapolation. The confidence interval for the presented data is given in blue; the predicted number of samples for an upper CI with 0.5 of the mean is given in red.

References

[1] von Luxburg U. A tutorial on spectral clustering. Statistics and Computing. 2007 Dec;17(4):395-416.

[2] Damle A, Minden V, Ying L. Simple, direct and efficient multi-way spectral clustering. Information and Inference: A Journal of the IMA. 2019;8(1):181-203.

[3] Villarroel M, Chaichulee S, Jorge J, Davis S, Green G, Arteta C, et al. Non-contact physiological monitoring of preterm infants in the Neonatal Intensive Care Unit. npj Digital Medicine. 2019 Dec;2(1):128.

[4] Silverman BW, Titterington DM. Minimum Covering Ellipses. SIAM Journal on Scientific and Statistical Computing. 1980;1(4):401-9. Available from: https://doi.org/10.1137/0901028.

[5] Welzl E. Smallest enclosing disks (balls and ellipsoids). In: Maurer H, editor. New Results and New Trends in Computer Science. Berlin, Heidelberg: Springer Berlin Heidelberg; 1991. p. 359-70.

[6] Lu W, Nystrom MM, Parikh PJ, Fooshee DR, Hubenschmidt JP, Bradley JD, et al. A semi-automatic method for peak and valley detection in free-breathing respiratory waveforms. Medical Physics. 2006;33(10):3634-6.

[7] Ruangsuwana R, Velikic G, Bocko M. Methods to extract respiration information from ECG signals. In: 2010 IEEE International Conference on Acoustics, Speech and Signal Processing; 2010. p. 570-3.

[8] Zong W, Heldt T, Moody GB, Mark RG. An open-source algorithm to detect onset of arterial blood pressure pulses. vol. 30; 2003. p. 259 262.

[9] Cenci A, Liciotti D, Frontoni E, Mancini A. Non-Contact Monitoring of Preterm Infants Using RGB-D Camera; 2015. .

[10] Cobos-Torres JC, Abderrahim M, Martínez-Orgado J. Non-Contact, Simple Neonatal Monitoring by Photoplethysmography. Sensors. 2018;18(12).

[11] Orphanidou C, Bonnici T, Charlton P, Clifton D, Vallance D, Tarassenko L. Signal-quality indices for the electrocardiogram and photoplethysmogram: derivation and applications to wireless monitoring. IEEE J Biomed Health Inform. 2015 May;19(3):832-8.

[12] Makowski D, Pham T, Lau ZJ, Brammer JC, Lespinasse F, Pham H, et al. NeuroKit2: A Python toolbox for neurophysiological signal processing. Behavior Research Methods. 2021 feb;53(4):1689-96. Available from: https://doi.org/10.3758/s13428-020-01516-y.

[13] Grafton A, Warnecke JM, Li M, He E, Thomson L, Beardsall K, et al. Neonatal pose estimation in the unaltered clinical environment with fusion of RGB, depth and IR images. npj Digital Medicine. 2025 Aug;8(1):539. Available from: https://doi.org/10.1038/s41746-025-01929-z.
